# Fungal diversity in canopy soil of silver beech, *Nothofagus menziesii* (Nothofagaceae)

**DOI:** 10.1371/journal.pone.0227860

**Published:** 2020-01-24

**Authors:** Andy R. Nilsen, Suliana E. Teasdale, Paul L. Guy, Tina C. Summerfield, David A. Orlovich

**Affiliations:** Department of Botany, University of Otago, Dunedin, New Zealand; University of California Berkeley, UNITED STATES

## Abstract

Adventitious roots in canopy soils associated with silver beech (*Nothofagus menziesii* Hook.f. (Nothofagaceae)) form ectomycorrhizal associations. We investigated the extent to which canopy ectomycorrhizal communities contribute to overall diversity of ectomycorrhizal fungi associated with silver beech. Hyphal ingrowth bags were buried for 12 months in canopy and terrestrial soils of five trees at one site. We used amplicon sequencing of the nuclear ribosomal internal transcribed spacer 2 region (ITS2) to assess diversity of both ectomycorrhizal and non-ectomycorrhizal OTUs in hyphal ingrowth bags. There was a significant difference in ectomycorrhizal fungal community diversity between the terrestrial and canopy hyphal ingrowth bag communities. Ectomycorrhizal community composition of the terrestrial and canopy environments was also significantly different. Some ectomycorrhizal taxa were significantly differentially represented in either the terrestrial or canopy environment. The hyphal ingrowth bags also accumulated non-ectomycorrhizal species. The non-ectomycorrhizal fungi also had significantly different diversity and community composition between the canopy and terrestrial environments. Like the ectomycorrhizal community, some non-ectomycorrhizal taxa were significantly differentially represented in either the terrestrial or canopy environment. The canopy soil microhabitat provides a novel environment for growth of ectomycorrhizal adventitious roots and enables the spatial partitioning of ectomycorrhizal and non-ectomycorrhizal fungal diversity in the forest.

## Introduction

Aerial soils that accumulate on branches of old-growth trees support a wide variety of life [1]. Canopy soils support epiphytes [2, 3], epiphyte mycorrhizas [4, 5], other soil microorganisms [6] and soil fauna [7]. Adventitious roots from the host may grow into canopy soil and, depending on the host species and other factors, these roots can be either arbuscular mycorrhizal [8], ectomycorrhizal [9, 10] or non-mycorrhizal [11]. Thus, canopy soils host rich, diverse and variable fungal communities. These complex soil communities parallel their terrestrial counterparts, but the spatial separation of canopy soils from the ground and the invariably higher organic matter content of canopy soils [10, 12] makes canopy soils distinctive.

Many studies have reported on the diversity of organisms associated with canopy soil. In New Zealand, very high diversity of vascular and non-vascular plants and lichens were associated with canopies of the trees *Dacrycarpus dacrydioides* (A.Rich.) de Laub. (Podocarpaceae) and *Nothofagus menziesii* Hook.f. (Nothofagaceae) [2]. Several chytrids and an oomycete were reported from canopy soil of *N*. *menziesii* [13], and myxomycete plasmodia and fruit bodies of 9 species were observed in cultures from canopy soil and litter of *D*. *dacrydioides* and *N*. *menziesii* [14, 15]. Adventitious roots in the canopy of old-growth *N*. *menziesii* trees were ectomycorrhizal with a wide range of ectomycorrhizal fungi [10].

Fewer studies have compared fungal communities between the canopy and terrestrial soils. In *Quercus copeyensis* C.H.Müll. (Fagaceae) in Costa Rica [11], adventitious roots in canopy soil were non-mycorrhizal but were heavily colonized by dark septate fungi and other endophytes, whereas terrestrial roots were colonized by ectomycorrhizal fungi and no endophytes. This contrasts with the situation in other ectomycorrhizal trees studied (e.g. *Fagus sylvatica* L. (Fagaceae) [9] and *N*. *menziesii* [10]), where the adventitious canopy roots were ectomycorrhizal. Canopy roots of *Fagus sylvatica* were less colonized by ectomycorrhizal fungi than were terrestrial roots (87% versus 93%, [9]).

In the present study, we compared the diversity of fungi in canopy soils of New Zealand old-growth silver beech, *N*. *menziesii*, with fungi in terrestrial soils adjacent to the same trees using hyphal ingrowth bags [16]. Studies have shown that hyphal ingrowth bags preferentially accumulate ectomycorrhizal fungi. For example, 88% of clones from hyphal ingrowth bags belonged to ectomycorrhizal fungal families in an Australian wet sclerophyll forest [17], and 83% of clones from hyphal ingrowth bags in a Danish beech (*F*. *sylvatica*) forest belonged to ectomycorrhizal species [18]. In the present study, hyphal ingrowth bags were buried in canopy and terrestrial soils and allowed to accumulate fungi for 12 months. While the primary focus of the present study was ectomycorrhizal fungi, we found that the bags also accumulated non-ectomycorrhizal fungi. Therefore, we analysed both ectomycorrhizal and non-ectomycorrhizal fungi separately in the hyphal ingrowth bags using DNA barcoding.

New Zealand is unique in having relatively few native ectomycorrhizal plant species [19], namely species of *Nothofagus* and the two myrtaceous genera *Leptospermum* and *Kunzea*. The epiphytes growing in the canopy of silver beech are not typically ectomycorrhizal [2], and rare *Nothofagus* seedlings growing as facultative epiphytes were not observed in this nor our previous study of canopy ectomycorrhizal communities [10], so those ectomycorrhizal fungi found in canopy soils are predominately associated with host adventitious roots and not the roots of epiphytes. Species of *Nothofagus* are obligately ectomycorrhizal [20], so it is of critical importance to understand the diversity of ectomycorrhizal fungi associated with *Nothofagus* as an aspect of management of *Nothofagus* forests. The hyphal ingrowth bag system allows us to compare communities of ectomycorrhizal fungi associated with roots of the same host tree but separated spatially by either growing in the terrestrial (low organic matter) or the canopy (high organic matter) communities. Such a system may be analogous to the situation were different rooting zones can harbour different communities of ectomycorrhizal fungi. For example, the community composition of ectomycorrhizal fungi in mineral soil rooting zones of the tropical tree *Dicymbe corymbosa* was significantly different to that in organic soil rooting zones, and was interpreted as evidence of niche partitioning amongst the ectomycorrhizal communities [21]. Canopy soil associated with old-growth silver beech forest at the present study site is high in organic matter (86% organic matter, compared with 10% organic matter in terrestrial soil [10]), the canopy soils are younger than the terrestrial soils. Given the large difference in organic matter content in canopy versus terrestrial soils in these forests, and the relative ages of the two environments, we predict that niche partitioning will be evident in this case as well, not only for ectomycorrhizal species, but non-ectomycorrhizal fungi in hyphal ingrowth bags as well.

The physical separation of the canopy soil from the terrestrial soil means that access to the canopy environment might be limited by dispersibility of propagules that originate from the terrestrial environment. For example, fungi that have wind-dispersed spores might have easier access to canopy soil than fungi that disperse by vegetative mycelial growth. Similarly, fungi that are dispersed by animals (e.g., grazing birds [22, 23], bats, lizards or insects [22] hypothesised to disperse sequestrate fungi in New Zealand) might have differing access to the canopy soil, depending on whether the dispersers themselves access the forest canopy. Thus, there are many reasons why the canopy fungal communities could differ in aspects of their diversity and composition between terrestrial and canopy communities. We test the hypotheses that (i) ectomycorrhizal composition will differ between terrestrial and canopy soils, (ii) that canopy soils host lower beta-diversity than terrestrial soils.

## Materials and methods

The New Zealand Department of Conservation granted permission to carry out the field work undertaken in this study. The Ngāi Tahu Research Consultation Committee at the University of Otago was consulted during the planning of this research.

### Field site description

**Fig 1:**
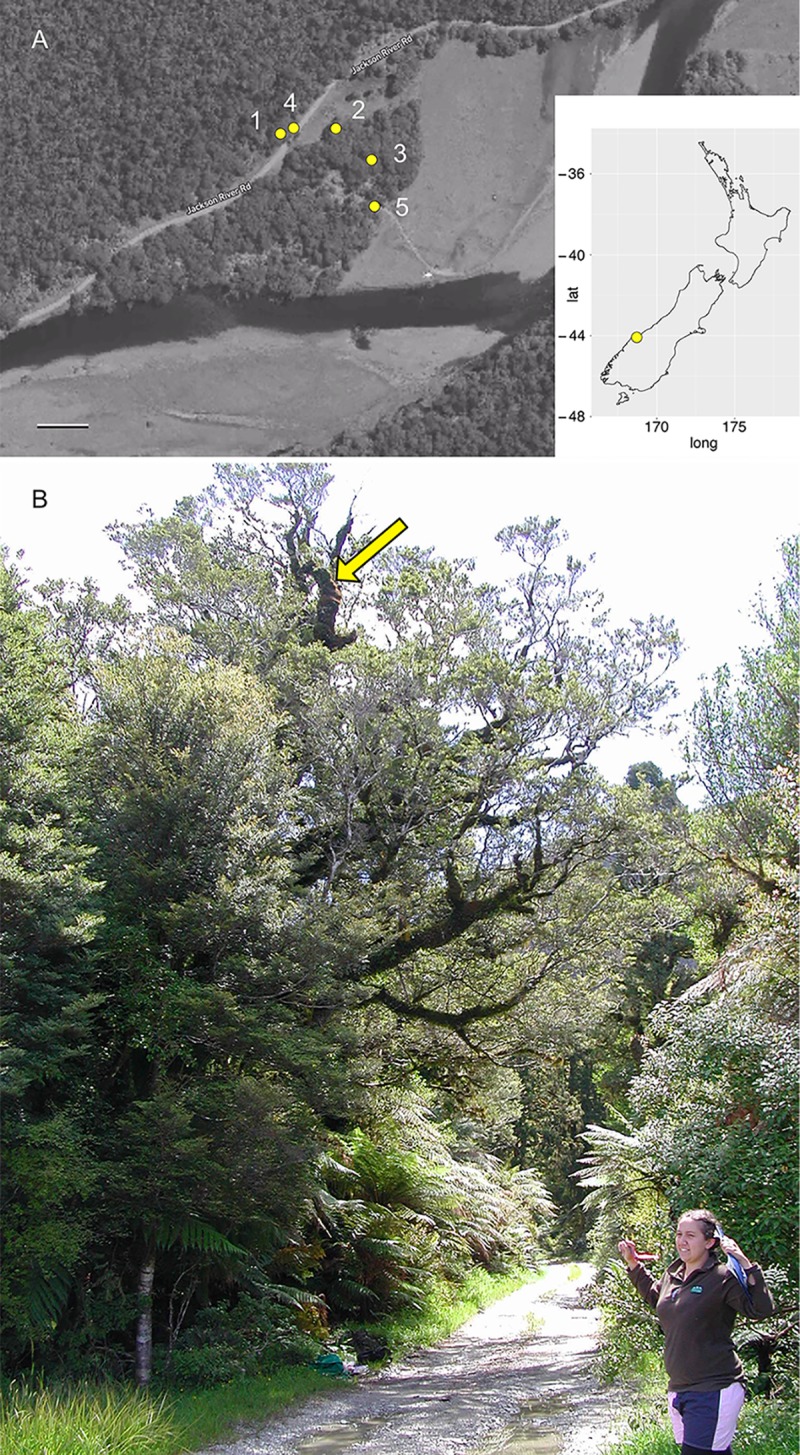
(A) Aerial photograph of the study site, indicating the position of each tree sampled. Tree numbers correspond to numbers in S1 Table. Scale = 50 m. Inset: Map of New Zealand showing the location of the study site. (B) Photograph of old-growth *Nothofagus menziesii* (tree 5, arrow) at the study site.

**Fig 2:**
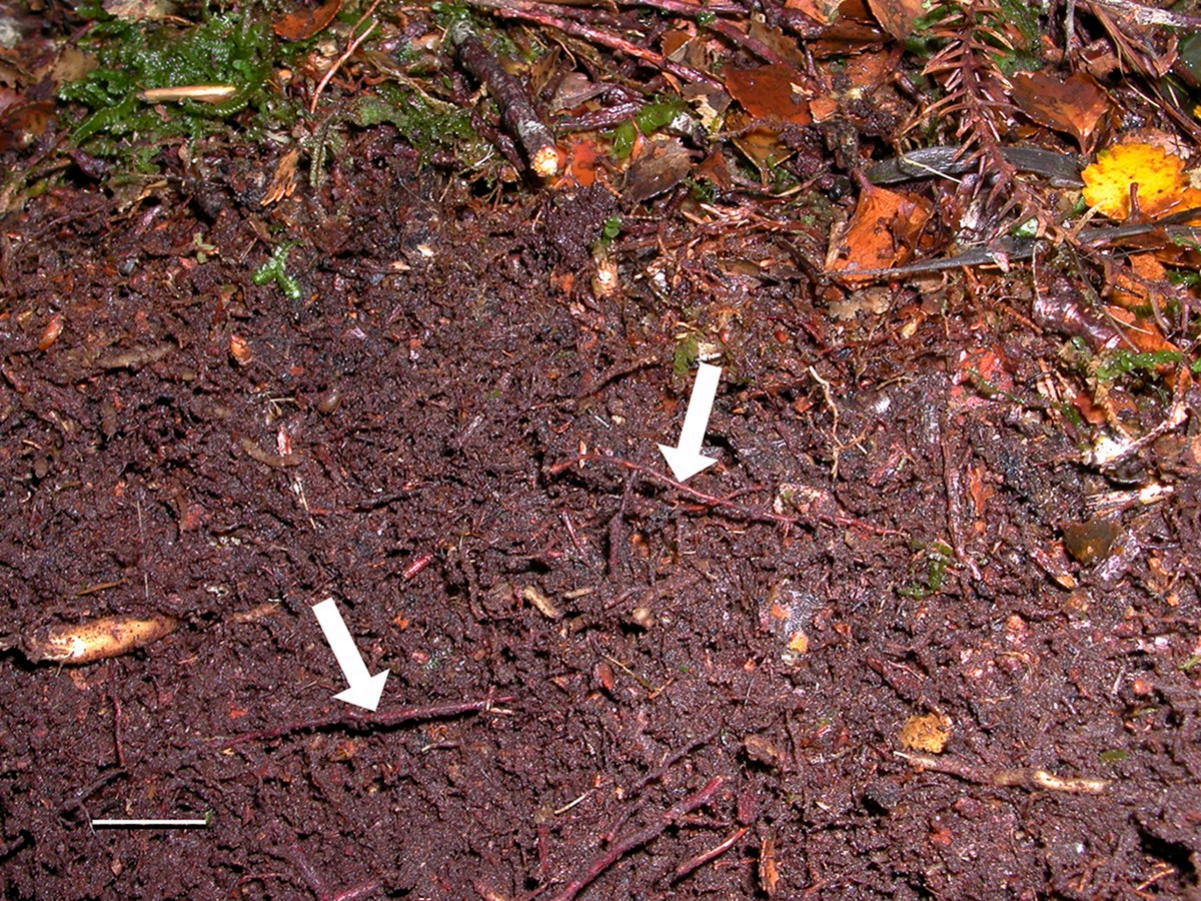
Photograph of canopy soil at the study site. The epiphyte layer has been removed to expose the organic matter and adventitious roots (arrows). Scale = 10 mm.

The field site was a mixed southern beech-podocarp cool-temperate rainforest ~ 2 km south of the junction of the Jackson and Arawhata Rivers, 33 km southwest of Haast, Southern Westcoast, New Zealand (latitude −44.055, longitude 168.709, Fig 1). The map reference for the site is BZ11 563130 (Map NZTopo50-BZ11 Mt Pollux 1:50,000). The mean air temperature at Haast (Haast Aws station, latitude −43.861, longitude 169.007) is 11.5°C (mean 2000–2001, 2003–2005, 2007–2015), and the mean annual rainfall is 3125 mm (mean annual rainfall average from years 2000, 2002–2016, 2011–2012) (data from CliFlo: NIWA's National Climate Database on the Web, http://cliflo.niwa.co.nz/, retrieved 1 December 2017). The site is significant because it is within Te Wāhipounamu: South West New Zealand World Heritage Area, an area with a long history of ecological and conservation research [24]. Five trees were selected for burial of hyphal ingrowth bags (Fig 1). Trees were selected on the basis of rope accessibility and safety for climbing to access branches with large accumulations of canopy soil. The location and dimension of each of the five trees sampled are given in S1 Table. The trees had extensive development of canopy soil on the branches (Fig 2). Based on a growth rate of 2.2 mm per year [25] and a diameter at breast height of > 1 m (S1 Table), the age of the trees is estimated at > 450 years, and while it is difficult to estimate the age of the canopy communities, it is expected that the most well-developed canopy soils are at least several hundred years old.

### Sample collection

Hyphal in-growth bags were constructed of 2 × 2 cm 50 μm nylon mesh containing 1.2 g of acid washed sand (Unilab, Australia). Five hyphal ingrowth bags were buried in terrestrial soil within the dripline of each of the five trees (S1 Table) and at least 1 m away from the base of the trunk. Each bag was buried ≥ 2 m apart at a depth of 4–5 cm. Five canopy bags were buried 4–5 cm deep in canopy soil of each tree, with each bag buried on a different branch (height above ground of each hyphal ingrowth bag is given in S1 Table, and ranged from 1.9–17.3 m above the ground). A total of 50 hyphal in-growth bags were buried in the canopy and terrestrial soils around the five selected trees. After a 12-month period the hyphal ingrowth bags were retrieved and kept at 4°C for no longer than 48 hours before freeze-drying. Adhering soil was removed from the outer surface of each hyphal ingrowth bag. The content of each bag was placed into individual mortars and frozen by covering with liquid nitrogen. The mortars were then wrapped with aluminium foil, placed into a vacuum chamber and freeze-dried for 14 h. Dried samples were placed in resealable bags containing silica gel and stored at −20°C until DNA extraction.

### DNA extraction and sequencing

Prior to DNA extraction, the freeze-dried sand samples were mixed using a sterile spatula. Environmental DNA in the hyphal in-growth bags was extracted from 0.25 g of the sand using the PowerSoil DNA Isolation Kit (MoBio, Bio-Strategy, Auckland, New Zealand) as per the manufacturer’s instructions. Isolated DNA from the hyphal ingrowth bags were diluted ten-fold before PCR.

Amplicons were generated in a two-step process for sequencing on the Illumina MiSeq platform. Amplification of the nuclear ribosomal DNA from the ITS2 region was performed using the fungal specific primer pair ITS3_KY02 [26] and ITS4 [27] with Illumina TruSeq adapter sequences (IDT, Singapore and Custom Science Ltd, New Zealand). Each 50 μL first-round PCR reaction contained 10 μL of 5 × KAPA HiFi HotStart buffer (KAPA Biosystems, USA), 10 nmol of dNTP mix, 17.5 pmol of ITS3_KYO2 and ITS4, 0.2 μL of 10% bovine serum albumin, 1 U KAPA HiFi HotStart DNA Polymerase, 2 μL of a 1:10 dilution of gDNA and sterile H_2_O q.s..

Following the initial denaturing step at 95°C for five minutes, 30 cycles of 98°C, 47°C and 72°C each for one minute, with a final extension step of 72°C for five minutes were performed on a thermocycler (Biometra TGradient, Göettingen, Germany). PCR products were visualized on 1.5% agarose gels.

Amplicons were purified using the Agencourt AMPure XP PCR purification system (Beckman Coulter, USA). DNA concentration was determined using a fluorimeter (Qubit, Invitrogen, USA) and the Qubit dsDNA HS assay kit. Amplicons were diluted to 1 ng/μL. Purified first-round PCR products were used as a template for second-round PCR. Each 50 μL reaction contained: 10 μL of 5 × KAPA HiFi HotStart buffer, 10 nmol of dNTP mix, 10 pmol each of primers with dual-indexed (i5 and i7) Illumina Nextera adapters, 1 U KAPA HiFi HotStart DNA polymerase, 2 ng of PCR product and sterile H_2_O q.s. Amplicons were visualized, purified and quantified as described above. Amplicon libraries were sequenced on the Illumina MiSeq using v2 chemistry allowing 250 bp paired-end reads by Otago Genomics Facility. Negative controls comprising water-only template PCRs and PCRs of the same acid-washed sand used in the hyphal ingrowth bags were also prepared and sequenced in the same manner.

### Bioinformatics

Paired-end reads were merged using USEARCH v11.0.667 [28] allowing a minimum of 97% similarity across the overlap. The samples were filtered at maximum expected errors (maxee) > 1.0 and the ITS2 variable region was extracted using ITSx v1.0.11 [29]. The reads were clustered at 97% using UPARSE and singletons were discarded [30]. OTUs were assigned taxonomy against the utax reference database 2.2.2019 using SINTAX [31] with a sintax_cutoff of 0.8, implemented in USEARCH. OTUs with high read counts (> 10 reads) in the control sample were deleted. This affected only four OTUs that were predominantly represented in the control sample. The samples were rarefied to the lowest reads per sample, 9,500 reads, in Qiime v1.9.1 [32], and separated into trophic guilds using FUNGuild v1.0 [33]. Guilds were combined into two groups: (i) ectomycorrhizal (using a strict criterion of selecting only the group ‘Ectomycorrhizal’ with a confidence rating of ‘Highly Probable’ or ‘Probable’) and (ii) non-ectomycorrhizal (comprising all other groups, excluding those OTUs that could not be assigned to functional guild). Because of the strict criterion for inclusion in the ectomycorrhizal group, it was expected that the non-ectomycorrhizal group would contain putative ectomycorrhizal OTUs with low confidence ratings. OTUs with an abundance of greater than 1% in either of the soil types that could not be assigned to functional group because of poor taxonomic assignment were searched against sequences in GenBank using BLAST [34] and reassigned in the FunGuild table (S2 Data). Those OTUs not assignable to any functional guild were excluded from further analysis. Identity of the top 25 most abundant ectomycorrhizal and non-ectomycorrhizal OTUs from both the canopy and terrestrial samples was cross-checked by conducting a search on UNITE [35] using massBLASTer. The species hypothesis corresponding to the sequences most similar to each OTU was selected. In some cases, we conducted closer phylogenetic exanimations of OTU sequences to determine the most accurate name where this disagreed with the most closely matching species hypothesis. Where no closely matching sequences could be named to species, we selected the species hypothesis from a higher taxonomic rank. A 1.5% threshold was selected for the most closely matching species hypothesis, unless a hypothesis at a lower threshold indicated different and more accurate identification.

The statistical analyses were performed in Qiime v1.9.1 and in R v3.3.2 using the packages phyloseq version 1.19.1 [36] and vegan version 2.4–2 [37]. The alpha diversity metrics: observed species, Simpson’s diversity and Shannon-Weiner diversity, were calculated and plotted in phyloseq. Evenness was calculated by dividing the Shannon diversity by the log_*n*_ of observed species. Because sample numbers differed in the terrestrial and canopy environments, each community was subsampled 1000 times to a maximum of 11 (the number of canopy samples) using resampling with replacement, calculating the mean for each replicate. Results were summarized by determining the mean, SD, median and range for each metric. The Mann-Whitney rank sum test, in the base R package, was used to test for statistical difference between alpha metrics. Beta diversity was calculated using Bray-Curtis dissimilarity and the differences between communities were visualized by non-metric multidimensional scaling (nMDS) in phyloseq. Adonis in vegan was used to test for differences in community composition between soil types, and betadisper was used to test for differences in dispersion of communities in each soil type by a permutation test for homogeneity of multivariate dispersions, with 999 permutations. Differential representation in the abundances of OTUs were tested using the Kruskal-Wallis test in Qiime.

Heat maps were generated in R with guidance from http://www.molecularecologist.com/2013/08/making-heatmaps-with-r-for-microbiome-analysis/ (accessed 7 April 2017) and using the packages: gplots version 3.0.1 [38], Heatplus version 2.20.0 [39], vegan version 2.4–2 and RColorBrewer version 1.1–2 [40]. The Bray-Curtis matrix was clustered using average linkage hierarchical clustering and only OTUs with a relative abundance of greater than 5% in at least one sample were displayed in the heat map. Clustering was performed on the OTUs and samples.

Sequence data were submitted to NCBI, BioProject PRJNA421209, BioSample accession numbers 8164397–8164428.

### Phylogenetic analysis

Most OTUs identified as ectomycorrhizal species were of Australasian origin, but one OTU (OTU112) was identified as *Hebeloma hiemale* Bres., a species introduced to New Zealand [41]. To confirm the identity of OTU112, we aligned that sequence with a selection of internal transcribed spacer sequences from a detailed study of *Hebeloma* section *Denudata* [42] and performed a phylogenetic analysis using Bayesian inference, following the method described in Rees et al. [43] but without coding indels. Species from subclade /mediorufum [43] were used as outgroups. The alignment and phylogeny are available from www.treebase.org, accession number 23169 (http://purl.org/phylo/treebase/phylows/study/TB2:S23169). [Review access URL: http://purl.org/phylo/treebase/phylows/study/TB2:S23169?x-access-code=b870be30134103a0d3cf7958d6c06df0&format=html]

## Results

### Hyphal ingrowth bag recovery

Thirteen of the original 25 hyphal ingrowth bags were recovered from the 5 tree canopies and 20 of 25 terrestrial samples were recovered after the 12-month incubation period. Those that were not recovered were either missing or were found unburied at the site. Two canopy samples were unsuccessfully amplified by PCR, with the remaining 31 samples successfully amplified and prepared for sequencing.

### OTU clustering and trophic guilds of OTUs recovered from hyphal ingrowth bags

Amplicon sequence clustering resulted in 6,136 OTUs (S1 Data), of which ~80% (4612 OTUs from 294,500 reads) were parsed by FUNGuild (Table 1). Of those parsed OTUs, 78% were assigned to a functional group (comprising 1,320 OTUs), and ~22% were not (3,292 OTUs).

**Table 1:** Number of reads after rarefaction of each sample to a depth of 9500 reads. ECM = ectomycorrhizal.

	Reads	OTUs	% total reads	% total OTUs
**No functional group**	64,970	3,292	22	71
**Non-ECM**	62,826	921	21	20
**ECM**	166,704	399	57	9
**Total**	294,500	4,612	100	100

Whilst the ECM fungal reads comprised 57% of the total reads across all samples (Table 1), the terrestrial samples had a greater (p < 0.001) proportion of ECM fungal reads (66%) than the canopy ECM fungal reads (39%) (Table 2). The non-ECM fungal reads were relatively less abundant (p < 0.001) in the terrestrial samples (14%) and more abundant (p < 0.001) in the canopy samples (34%) (Table 2). Those fungal reads not assigned to a functional group comprised 20% of the terrestrial reads and 26% of the canopy reads (Table 2).

**Table 2:** Mean proportion of total reads assigned to functional groups in canopy and terrestrial samples. ECM = ectomycorrhizal.

		Mean proportion of total reads	SD	Median	Range	Wilcoxon W, p-value
**ECM**						
	**Terrestrial**	0.66	0.06	0.66	0.47–0.81	4387.5, < 0.001
	**Canopy**	0.39	0.08	0.39	0.15–0.66	
**Non-ECM**						
	**Terrestrial**	0.14	0.04	0.14	0.06–0.27	997630, < 0.001
	**Canopy**	0.34	0.06	0.34	0.18–0.55	
**No functional group**						
	**Terrestrial**	0.20	0.04	0.20	0.11–0.35	877870, < 0.001
	**Canopy**	0.26	0.04	0.26	0.15–0.38	

### Community diversity

The terrestrial ECM fungal communities were richer (p < 0.001) but less even (p < 0.001) than the canopy ECM communities, and the terrestrial ECM fungal communities had slightly higher Simpson (p < 0.001) and lower Shannon (p < 0.001) diversities (Table 3). Non-ECM fungal communities were richer (p < 0.001) and more even (p < 0,001) in the terrestrial environment, and the terrestrial non-ECM fungal communities had higher Simpson (p < 0.001) and Shannon (p < 0.001) diversities (Table 3).

**Table 3:** Diversity parameters of ectomycorrhizal and non-ectomycorrhizal OTUs in canopy and terrestrial communities, from 1000 replicate bootstrap analyses with replacement, sampling 11 samples at random per replicate.

		Mean	SD	Median	Range	Wilcoxon W, p-value^a^
**Ectomycorrhizal OTUs**						
**Observed OTUs**						
	**Terrestrial**	83.06	9.86	82.45	56.09–115.55	141600, < 0.001
	**Canopy**	70.88	5.91	70.82	52.82–91.00	
**Evenness**						
	**Terrestrial**	0.45	0.04	0.45	0.35–0.62	828690, < 0.001
	**Canopy**	0.54	0.08	0.54	0.29–0.78	
**Simpson (1-D)**						
	**Terrestrial**	0.72	0.04	0.71	0.61–0.82	401920, < 0.001
	**Canopy**	0.69	0.10	0.68	0.40–0.94	
**Shannon**						
	**Terrestrial**	1.98	0.24	1.96	1.39–2.78	757510, < 0.001
	**Canopy**	2.28	0.36	2.28	0.83–3.30	
**Non-ectomycorrhizal OTUs**						
**Observed OTUs**						
	**Terrestrial**	125.36	13.67	125.45	89.91–166.09	23532, < 0.001
	**Canopy**	98.68	5.85	97.09	79.36–113.27	
**Evenness**						
	**Terrestrial**	0.68	0.03	0.68	0.55–0.77	70810, < 0.001
	**Canopy**	0.54	0.05	0.54	0.33–0.69	
**Simpson (1-D)**						
	**Terrestrial**	0.88	0.02	0.88	0.79–0.95	10429, < 0.001
	**Canopy**	0.76	0.06	0.76	0.51–0.89	
**Shannon**						
	**Terrestrial**	3.24	0.15	3.24	2.79–3.74	1695, < 0.001
	**Canopy**	2.45	0.24	2.46	1.66–3.13	

^a^Results of Wilcoxon rank sum test between canopy and terrestrial samples.

### Community composition

**Fig 3:**
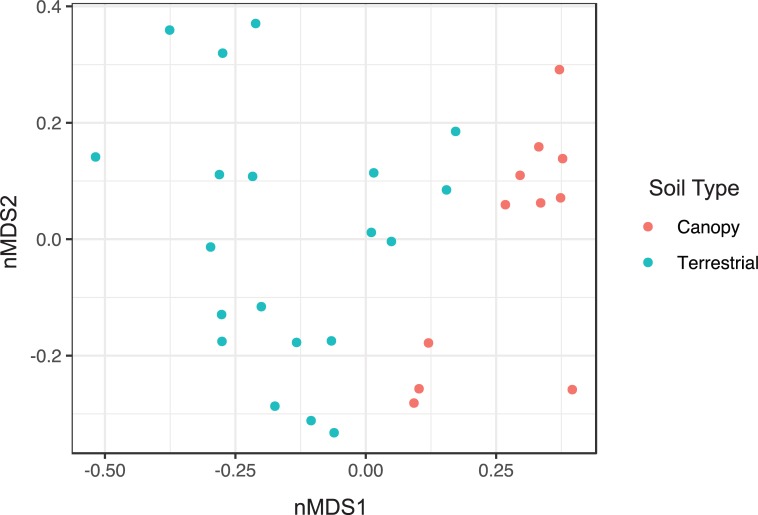
Ordination by non-metric multidimensional scaling of ectomycorrhizal OTUs, using Bray-Curtis dissimilarity. Stress: 0.1527015. Teal circles: canopy samples; red circles: terrestrial samples.

**Fig 4:**
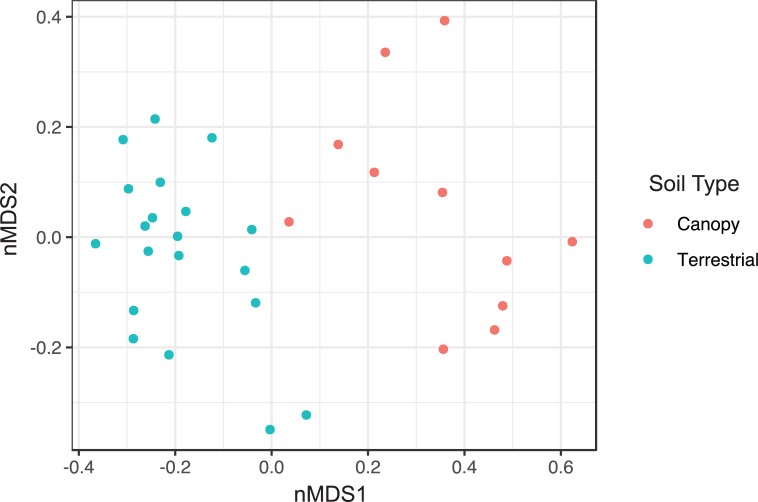
Ordination by non-metric multidimensional scaling of non-ectomycorrhizal OTUs, using Bray-Curtis dissimilarity. Stress: 0.1488418. Teal circles: canopy samples; red circles: terrestrial samples.

Terrestrial fungal communities were different in composition from canopy fungal communities in both ectomycorrhizal (Fig 3) and non-ectomycorrhizal (Fig 4) fungal groups. In both cases, the canopy samples were associated with one side of the ordination space and the terrestrial samples the other side. The centroids of terrestrial and canopy fungal communities were significantly different for both ectomycorrhizal (P = 0.001, S2 Table) and non-ectomycorrhizal (P = 0.001, S4 Table) groups by the PERMANOVA test. The dispersions of samples in each soil type were not significantly different (ectomycorrhizal: P = 0.062, S3 Table; non-ectomycorrhizal: P = 0.434, S5 Table), so the difference between canopy and terrestrial fungal communities is interpreted to be due to community composition.

### Dominant ectomycorrhizal fungi in terrestrial and canopy communities

Analysis of the terrestrial and canopy samples revealed a diverse array of ectomycorrhizal fungi (Table 4). The most abundant ectomycorrhizal OTU in terrestrial samples (OTU2) was identified as *Cortinarius thaumastus* and comprised 13.3% of reads in the terrestrial samples. The most abundant ectomycorrhizal OTU in canopy samples (OTU1) was identified as *Laccaria violaceonigra*, comprising 38.8% of the canopy reads. *Laccaria violaceonigra* (OTU1) was also the second most abundant OTU in the terrestrial samples, comprising 12.4% of the reads in that environment. Six OTUs in the terrestrial samples had a relative abundance ≥ 5% and 18 had a relative abundance ≥ 1%. Four OTUs in the canopy samples had a relative abundance ≥ 5% and 14 had a relative abundance ≥ 1%. Diversity of *Cortinarius* differed between terrestrial and canopy samples, with 4 OTUs identified as *Cortinarius* amongst the top 25 OTUs in terrestrial samples, compared to 15 OTUs identified as *Cortinarius* amongst the top 25 OTUs in canopy samples.

**Table 4:** Top 25 OTUs of ectomycorrhizal species in terrestrial and canopy samples, ranked by the total number of reads. The name, species hypothesis (SH) and reference sequence are given for the closest matching sequence on UNITE. MisM = number of nucleotide mismatches between the query (OTU) and reference sequences; Q start/Q end = 5′/3′ base positions of the OTU sequences; R start/R end = 5′/3′ base positions of the reference sequences.

**OTU**	**Count**	**Relative abundance**	Reference	Most similar species hypothesis (SH)	SH name	Percent identity	MisM	Qstart	Qend	Rstart	Rend	Origin of reference sequence
**Terrestrial**												
OTU2	16693	0.13296957	JQ287673	SH2124709.08FU	*Cortinarius thaumastus*	100	0	1	181	446	626	NZ
OTU1	15524	0.1236578	KU685710	SH2252839.08FU	*Laccaria violaceonigra*	100	0	1	205	438	642	NZ
OTU3	12579	0.10019914	DQ672324	SH1528514.08FU	Thelephoraceae	96.82	6	1	220	395	613	Australia
OTU7	11310	0.09009081	UDB002698	-	Envir: Cantharellaceae	94.65	3	3	187	521	702	Australia
OTU4470	8189	0.06523021	JX648601	SH1504088.08FU	*Cortinarius*	98.9	1	1	180	440	620	NZ
OTU6	7721	0.06150231	UDB004029	SH1528630.08FU	Envir: Thelephoraceae	96.35	7	1	218	365	583	Australia
OTU13	4510	0.0359248	EF634121	SH1546109.08FU	*Clavulina*	98.76	2	1	240	413	653	NZ
OTU14	4429	0.03527959	KF871770	SH1562311.08FU	*Inocybe*	93.86	6	1	220	462	689	Australia
OTU12	3917	0.03120121	GU222261	SH2272053.08FU	*Russula tricholomopsis*	99.64	1	1	274	404	677	NZ
OTU10	3735	0.02975147	KY684373	SH1650399.08FU	Cantharellaceae	96.11	6	1	180	571	749	China
OTU28	2165	0.0172455	JX625359	SH1502583.08FU	Thelephoraceae	94.62	9	1	223	365	584	Italy
OTU387	2165	0.0172455	GU222307	SH1546157.08FU	*Clavulina*	98.33	4	1	240	418	657	NZ
OTU43	1781	0.01418671	JQ279512	SH2528746.08FU	*Lactarius*	100	0	1	263	416	678	NZ
OTU24	1718	0.01368488	MH019833	SH1551663.08FU	Fungi	89.17	21	1	238	384	620	Argentina
OTU18	1629	0.01297594	UDB014880	-	Envir: Pezizales	98.88	2	1	178	108	285	NZ
OTU35	1586	0.01263342	KY462407	SH1651300.08FU	*Inocybe*	90.3	5	23	149	433	565	Chile
OTU20	1581	0.0125936	KP636873	SH1562206.08FU	*Astrosporina subclavata*	97.85	1	1	184	333	517	NZ
OTU31	1264	0.0100685	JX316439	SH2544936.08FU	*Cenococcum geophilum*	100	0	1	146	311	456	Argentina
OTU40	1254	0.00998885	GU222324	SH2272056.08FU	*Russula roseostipitata*	100	0	1	272	384	655	NZ
OTU26	1105	0.00880198	KU523937	SH2147886.08FU	*Descolea gunnii*	100	0	1	209	456	664	NZ
OTU29	1085	0.00864266	UDB014331	SH1504007.08FU	Envir: Cortinariaceae	96.12	7	1	205	408	613	Argentina
OTU3083	1020	0.0081249	GU222307	SH1546157.08FU	*Clavulina*	96.67	8	1	240	418	657	NZ
OTU5000	1003	0.00798949	UDB004029	SH1528630.08FU	Envir: Thelephoraceae	96.35	7	1	218	365	583	Australia
OTU27	918	0.00731241	MG019344	SH2122097.08FU	*Cortinarius*	99	2	1	201	463	663	NZ
OTU48	891	0.00709734	MF461604	SH2310360.08FU	*Russula griseobrunnea*	100	0	1	226	417	642	NZ
**Canopy**												
OTU1	15968	0.38791177	KU685710	SH2252839.08FU	*Laccaria violaceonigra*	100	0	1	205	438	642	NZ
OTU8	3910	0.09498591	KY774032	SH1546155.08FU	*Clavulina*	95.85	9	1	241	420	659	New Caledonia
OTU1127	3225	0.07834516	EF634117	SH2253408.08FU	*Laccaria*	99.51	1	1	205	442	646	NZ
OTU16	3107	0.07547857	EF634088	SH1528436.08FU	Thelephoraceae	100	0	1	216	407	622	NZ
OTU27	1516	0.0368283	MG019344	SH2122097.08FU	*Cortinarius*	99	2	1	201	463	663	NZ
OTU226	1319	0.03204256	UDB004029	SH1528630.08FU	Envir: Thelephoraceae	95.43	10	1	219	365	583	Australia
OTU55	975	0.02368574	MH101610	SH1504292.08FU	*Cortinarius cucumeris*	98.54	0	1	203	452	657	NZ
OTU5000	954	0.02317559	UDB004029	SH1528630.08FU	Envir: Thelephoraceae	96.35	7	1	218	365	583	Australia
OTU59	919	0.02232533	MG552976	SH2122659.08FU	*Cortinarius*	99.5	0	1	198	402	600	Australia
OTU37	911	0.02213099	KP191825	SH2288501.08FU	*Austropaxillus macnabbii*	100	0	1	209	416	624	NZ
OTU80	848	0.02060052	KT334128	SH2123685.08FU	*Cortinarius porphyroideus*	100	0	1	201	448	648	NZ
OTU41	730	0.01773394	KJ635245	SH1503938.08FU	*Cortinarius orixanthus*	95.1	9	1	203	449	652	NZ
OTU56	548	0.0133126	KJ635239	SH2122019.08FU	*Cortinarius veronicae*	100	0	1	201	450	650	NZ
OTU75	468	0.01136916	LT000117	SH1647807.08FU	*Tricholoma viridiolivaceum*	100	0	1	201	403	603	NZ
OTU61	396	0.00962006	KY462421	SH1504760.08FU	*Cortinarius*	91.15	14	1	189	408	599	Argentina
OTU126	359	0.00872121	MH101550	SH2123955.08FU	*Cortinarius rotundisporus*	100	0	1	202	446	647	NZ
OTU83	315	0.00765232	JQ282169	SH1504725.08FU	*Cortinarius*	97	3	1	198	454	652	NZ
OTU136	248	0.00602468	MH101523	SH2586004.08FU	*Cortinarius*	100	0	1	134	341	474	NZ
OTU147	230	0.00558741	KC017360	SH2121746.08FU	*Cortinarius*	99.5	1	1	202	404	605	NZ
OTU89	222	0.00539306	UDB004041	SH1606335.08FU	Envir: Clavulinaceae	99.15	0	1	232	358	591	Australia
OTU112	210	0.00510155	JX178629	SH2291742.08FU	*Hebeloma hiemale*	100	0	1	215	443	657	NZ
OTU134	159	0.0038626	MH101581	SH2122340.08FU	*Cortinarius*	100	0	1	199	421	619	NZ
OTU111	158	0.00383831	JF960721	SH1504362.08FU	*Cortinarius*	96.1	6	1	205	417	619	Australia
OTU113	155	0.00376543	MH101552	SH2121588.08FU	*Cortinarius wallacei*	100	0	1	203	441	643	NZ
OTU140	146	0.00354679	DQ328216	SH2121848.08FU	*Cortinarius*	100	0	3	201	436	634	Australia

Amongst the top 25 terrestrial and top 25 canopy ectomycorrhizal OTUs combined, there were 47 unique OTUs, of which 29 (62%) most closely matched sequences from New Zealand-collected material in GenBank, 10 OTUs (21%) matched sequences from Australian material, and 6 OTUs (13%) matched other Southern Hemisphere material. Most OTUs that matched sequences of named species in GenBank had very high identity (≥ 99%) to those sequences, and they were predominantly New Zealand endemic or Australasian species. However, OTU112 was identical to a sequence of *Hebeloma hiemale* (JX178629, Table 4), a species likely introduced to New Zealand from the Northern Hemisphere. Phylogenetic analysis (S1 Fig) indicated that this OTU is nested within other collections of *H*. *hiemale*, confirming this identification.

### Dominant non-ectomycorrhizal fungi in terrestrial and canopy communities

Analysis of the terrestrial and canopy samples revealed a diverse array of non-ectomycorrhizal fungi (Table 5). The most abundant non-ectomycorrhizal OTU in terrestrial samples (OTU11) was identified as *Mortierella humilis* and comprised 13% of reads in the terrestrial samples. The most abundant non-ectomycorrhizal OTU in canopy samples (OTU5) was identified as an unknown fungus identical to a sequence from *Pinus radiata* forest in New Zealand, comprising 33% of the canopy reads. This sequence had ~ 90–94% identity with sequences from the family Ceratobasidiaceae. Four OTUs in the terrestrial samples had a relative abundance ≥ 5% and 15 had a relative abundance ≥ 1%. Four OTUs in the canopy samples had a relative abundance ≥ 5% and 13 had a relative abundance ≥ 1%. Amongst the top 25 terrestrial and top 25 canopy non-ectomycorrhizal OTUs combined, there were 46 unique OTUs, of which only 7 (22%) most closely matched sequences from New Zealand-collected material in GenBank, 3 OTUs (9%) matched sequences from Australian material, 6 OTUs (19%) matched other Southern Hemisphere or equatorial (Colombian) material and 16 OTUs (50%) matched sequences of Northern Hemisphere material.

**Table 5:** Top 25 OTUs of non-ectomycorrhizal species in terrestrial and canopy samples, ranked by the total number of reads. The name, species hypothesis (SH) and reference sequence are given for the closest matching sequence on UNITE. MisM = number of nucleotide mismatches between the query (OTU) and reference sequences; Q start/Q end = 5′/3′ base positions of the OTU sequences; R start/R end = 5′/3′ base positions of the reference sequences.

OTU	count	Relative abundance	Reference	Most similar species hypothesis (SH)	SH name	Prcnt	MisM	Qstart	Qend	Rstart	Rend	Origin of reference sequence
**Terrestrial**												
OTU11	3568	0.1332	MG052956	SH2444324.08FU	*Mortierella humilis*	100	0	1	238	318	555	USA
OTU19	2959	0.1105	MH452344	-	Fungi	94.39	10	1	213	56	268	USA
OTU30	1463	0.0546	MG938353	SH2312004.08FU	*Nadsonia starkeyi-henricii*	100	0	1	166	384	549	Germany
OTU51	1350	0.0504	KX640357	SH2266986.08FU	*Mortierella*	100	0	1	248	324	571	Germany
OTU47	1242	0.0464	KY558367	SH2574334.08FU	*Solicoccozyma terricola*	100	0	1	234	390	623	Czechia
OTU44	863	0.0322	KX222781	-	Fungi	100	0	1	263	303	41	NZ
OTU32	716	0.0267	KU569541	SH2262523.08FU	*Ganoderma australe*	100	0	1	197	517	713	Brazil
OTU67	574	0.0214	MH633986	SH2266968.08FU	*Mortierella*	100	0	1	251	263	513	Spain
OTU60	574	0.0214	JX270502	SH2480509.08FU	*Apiotrichum*	100	0	1	164	335	498	US
OTU91	439	0.0164	AM999691	SH2298633.08FU	*Coprinopsis*	100	0	1	203	400	602	Norway
OTU125	360	0.0134	DQ403803	SH1608830.08FU	*Stephanospora redolens*	97.31	4	1	258	417	675	-
OTU79	334	0.0125	KX195252	-	*Ascocoryne*	94.44	8	1	144	109	252	US
OTU119	311	0.0116	MG916077	-	Fungi	99.21	0	1	251	1	251	-
OTU73	293	0.0109	KY750507	SH2303529.08FU	*Trichoderma polysporum*	100	0	1	166	415	580	-
OTU144	286	0.0107	KP311421	SH2267003.08FU	*Mortierella*	99.6	1	1	252	357	608	Australia
OTU215	265	0.0099	JN017915	SH2141209.08FU	*Armillaria novae-zelandiae*	100	0	1	268	479	746	NZ
OTU96	263	0.0098	JX975915	SH2269093.08FU	*Mortierella globulifera*	100	0	1	234	274	507	UK
OTU17	256	0.0096	X93980	SH2303512.08FU	*Trichoderma viride*	100	0	1	175	390	564	Germany
OTU129	255	0.0095	EF029209	SH1523256.08FU	*Chalara dualis*	97.95	3	1	146	336	481	-
OTU88	238	0.0089	JN628205	-	*Pilidium acerinum*	98.32	1	1	119	248	365	China
OTU38	233	0.0087	JX976028	SH1557049.08FU	*Mortierella zonata*	99.59	1	1	245	302	546	Colombia
OTU156	222	0.0083	KX222321	-	Fungi	90.21	6	1	135	183	41	NZ
OTU97	220	0.0082	MH711991	SH1594431.08FU	*Metarhizium anisopliae*	100	0	1	175	304	478	-
OTU110	208	0.0078	NR:073209	SH1616871.08FU	*Apiotrichum porosum*	100	0	1	163	306	468	-
**Canopy**												
OTU5	11993	0.3328	KX222388	-	Fungi	94.71	10	1	226	268	43	NZ
OTU15	3615	0.1003	MG020711	SH2268660.08FU	*Naganishia albida*	100	0	1	219	316	534	-
OTU17	2842	0.0789	X93980	SH2303512.08FU	*Trichoderma viride*	100	0	1	175	390	564	Germany
OTU38	2012	0.0558	JX976028	SH2267512.08FU	*Mortierella zonata*	99.59	1	1	245	302	546	Colombia
OTU21	1423	0.0395	EU552153	SH1614513.08FU	*Pyrenochaeta inflorescentiae*	98.2	2	1	167	521	686	South Africa
OTU36	1345	0.0373	JX316484	SH1561882.08FU	*Sebacina*	94.15	11	1	204	348	552	Argentina
OTU32	918	0.0255	KU569541	SH2262523.08FU	*Ganoderma australe*	100	0	1	197	517	713	Brazil
OTU45	839	0.0233	KU063815	-	Agaricomycetes	91.19	12	1	193	75	262	-
OTU82	597	0.0166	KP897191	-	*Vishniacozyma*	100	0	1	139	131	269	Lithuania
OTU50	586	0.0163	JX976121	SH2267026.08FU	*Mortierella gemmifera*	100	0	1	256	313	568	Netherlands
OTU123	558	0.0155	KP900722	SH1506670.08FU	*Mucor*	98.84	2	1	172	283	454	-
OTU72	401	0.0111	GU559986	SH2444871.08FU	*Mortierella fimbricystis*	100	0	1	238	283	520	Argentina
OTU58	395	0.0110	KM199341	SH2289955.08FU	*Pestalotiopsis arceuthobii*	100	0	1	163	388	550	US
OTU86	353	0.0098	DQ485645	SH1511111.08FU	*Terramyces*	98.38	3	1	185	341	525	-
OTU121	290	0.0080	MH651556	SH2232210.08FU	*Didymella macrostoma*	99.35	1	1	154	302	455	Russian Federation
OTU81	283	0.0079	MH753702	SH2272412.08FU	*Rhodotorula diobovata*	100	0	1	211	328	538	-
OTU133	270	0.0075	MG162216	-	Helotiales	94.2	6	1	138	21	156	-
OTU90	221	0.0061	UDB002743	SH1615427.08FU	Envir: *Sebacina*	98.99	2	1	198	323	520	Australia
OTU108	217	0.0060	MF976111	-	Fungi	96.45	4	1	196	66	260	NZ
OTU11	216	0.0060	MG052956	SH2444324.08FU	*Mortierella humilis*	100	0	1	238	318	555	US
OTU265	213	0.0059	MG915522	-	Fungi	93.18	11	2	177	1	175	-
OTU100	206	0.0057	JN225904	SH2290374.08FU	*Cylindrium*	100	0	1	148	380	527	NZ
OTU149	204	0.0057	JN225946	SH1548177.08FU	*Torrendiella brevisetosa*	100	0	1	147	347	493	NZ
OTU263	166	0.0046	JN206398	SH1522258.08FU	*Umbelopsis isabellina*	99.49	1	1	195	344	538	Colombia
OTU1919	155	0.0043	UDB002743	SH1615427.08FU	Envir: Sebacina	95.96	8	1	198	323	520	Australia

### Sample-to-sample diversity in hyphal ingrowth bags

**Fig 5:**
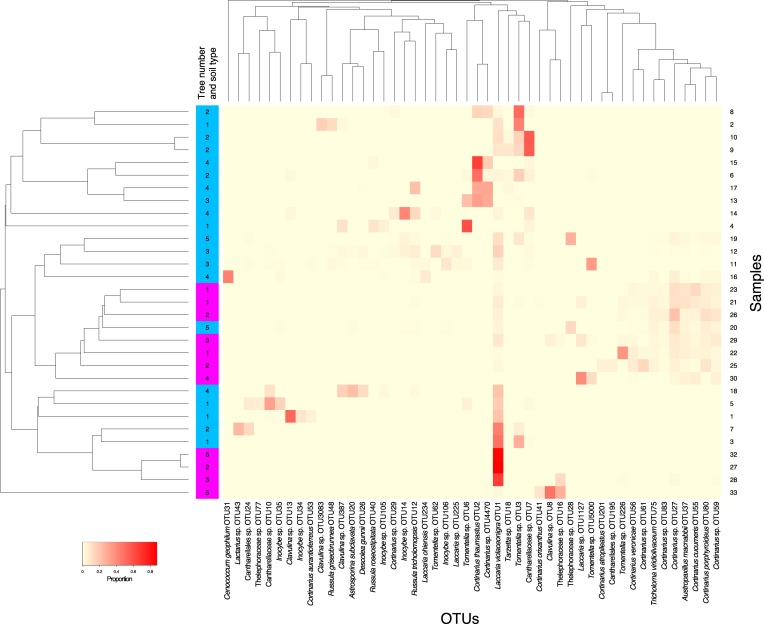
Heat map of ectomycorrhizal fungi in terrestrial (blue) and canopy (pink) hyphal ingrowth bags. Samples and OTUs are clustered based on Bray-Curtis dissimilarity. Relative abundance is indicated by the depth of colour.

**Fig 6:**
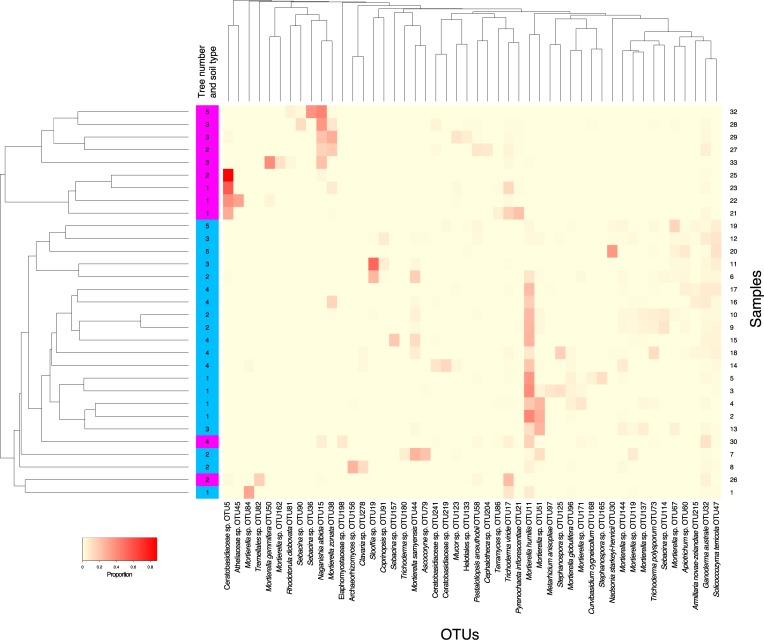
Heat map of non-ectomycorrhizal fungi in terrestrial (blue) and canopy (pink) hyphal ingrowth bags. Samples and OTUs are clustered based on Bray-Curtis dissimilarity. Relative abundance is indicated by the depth of colour.

Displaying the sample–sample diversity as heat maps of ectomycorrhizal (Fig 5) and non-ectomycorrhizal (Fig 6) fungi illustrates the patchiness of distribution of abundant OTUs.

When the ectomycorrhizal OTUs were clustered using Bray-Curtis dissimilarity (Fig 5), the samples do not cluster into canopy and terrestrial groups. OTU2 (*Cortinarius thaumastus*) was the most abundant terrestrial ectomycorrhizal OTU, but it was only present at high relative abundance in 5 of the terrestrial samples (collected from around the base of three different trees, Fig 5). Likewise, the dominant ectomycorrhizal OTU in canopy samples (OTU1, *Laccaria violaceonigra*) was only present at high relative abundance in some canopy samples, in addition to some terrestrial samples. There was no obvious relationship between Bray-Curtis dissimilarity of ectomycorrhizal samples and the tree from which each sample was taken (Fig 5).

The most abundant non-ectomycorrhizal OTU (OTU11, *Mortierella humilis*) was present at high relative abundance in most of the terrestrial samples (Fig 6). Canopy samples were either dominated by OTU5, which has similarity to the family Ceratobasidiaceae or OTU15, identified as the cryptococcal yeast *Naganishia albida*.

### Differential representation of fungi in canopy and terrestrial samples

Eight ectomycorrhizal OTUs had significantly greater relative abundance in terrestrial samples than canopy samples (S6 Table), when tested by the Kruskal-Wallis test using the conservative Bonferroni-corrected *p*-value. These OTUs, identified as *Descomyces* sp. (OTU49), *Clavulina* sp. (OTU387), *Laccaria ohiensis* (OTU234), two unidentified species of *Cortinarius* (OTU29 and OTU4470), *Cortinarius thaumastus* (OTU2), Cantharellaceae (OTU7) and *Inocybe arthrocystis* (OTU76), were very rare in canopy samples. Conversely, the ectomycorrhizal Thelephoraceae (OTU16) was significantly more abundant in canopy samples than in terrestrial samples. When the Kruskal-Wallis test was relaxed to use the false discovery rate (FDR)-corrected *p*-value, many more ectomycorrhizal OTUs were differentially represented (S6 Table), with 36 OTUs (55%) having greater representation in the terrestrial samples, and 30 OTUs (45%) having greater representation in canopy samples.

One non-ectomycorrhizal OTU (*Mortierella gamsii*, OTU144) was significantly represented in the terrestrial environment, when tested by the Kruskal-Wallis test using the conservative Bonferroni-corrected *p*-value (S7 Table). Four non-ectomycorrhizal OTUs were significantly differentially represented in the canopy (*Naganishia* sp. (OTU15), *Exobasidium* sp. (OTU265), *Penicillium* sp. (OTU411) and Bionectriaceae sp. (OTU196)) by the same criteria. Using the false discovery rate-corrected *p*-value (S7 Table), 11 OTUs (37%) had greater representation in the terrestrial samples, and 19 OTUs (63%) had greater representation in canopy samples.

## Discussion

The hyphal ingrowth bags accumulated fungi during the 12-month incubation period, a large proportion of which were identified as ectomycorrhizal taxa. The previously reported occurrence of adventitious canopy roots of the host trees [10] and the occurrence of mostly non-ectomycorrhizal ectomycorrhizal epiphytic plant species at the site [2] mean that the ectomycorrhizal fungi found in the canopy hyphal ingrowth bags are most likely predominantly associated with the host tree itself. However, we can’t exclude the possibility that some ectomycorrhizal fungi are associated with ectomycorrhizal epiphytes in the genera *Nothofagus*, *Leptospermum* or *Kunzea*. The discovery here of *Cortinarius rotundisporus* (OTU126) in the canopy hyphal ingrowth bags lends support to the idea that there were in fact myrtaceous host trees growing as epiphytes, given that this fungal species associates only with *Leptospermum* and *Kunzea* and not *Nothofagus* [44].

Both aerial and terrestrial soils associated with *Nothofagus menziesii* are host to diverse communities of fungi, although the canopy soil communities of ectomycorrhizal and non-ectomycorrhizal fungi being less rich than the terrestrial communities. he composition of the ectomycorrhizal community was different in each environment, with many species differentially represented to some degree in canopy or terrestrial communities. The finding that several ectomycorrhizal OTUs were significantly more represented in the terrestrial soil, whereas (under the Bonferroni-corrected *p*-value) only one OTUs had significantly greater representation in the canopy, could be explained by the canopy being less accessible to some species, or by each habitat being more or less suitable for those species. When the Kruskal-Wallis test was relaxed to use the FDR-corrected *p*-values, many OTUs were found to be differentially represented in both habitats, evidence that overall, the canopy soil increases habitat diversity for ectomycorrhizal species. Thus, the canopy soil represents a unique and additional, albeit slightly less-rich habitat for ectomycorrhizal fungi in this old-growth forest. By starting with bags containing only acid-washed sand, the technique allows the sampling of accumulated fungi that actively grew into the bags over the period of incubation. While the community of fungi in the hyphal ingrowth bags may be different to that detected by other methods (e.g. [18]), it serves a valuable comparative purpose.

It was expected to find non-ectomycorrhizal fungi in the hyphal ingrowth bags. Initially lacking carbon, the bags would have slowly accumulated carbon as fungi grew into the bags and subsequently died, providing a carbon source for later inhabitants of the bags. The hyphal ingrowth bags in the present study were incubated in situ for 12 months, so it is plausible that senescence of early colonising fungi would have occurred. Despite not being the target guilds of the study, it is notable that the non-ectomycorrhizal fungal communities also differed between canopy and terrestrial habitats, being less rich in the canopy than the terrestrial environment (the same pattern as the ectomycorrhizal fungal communities), and less even than the terrestrial communities (the opposite situation to the ectomycorrhizal fungal communities). Interestingly, while more ectomycorrhizal OTUs were significantly more represented in the terrestrial environment (55% of the differentially represented OTUs) than the canopy (45% of the OTUs), the non-ectomycorrhizal species showed the opposite pattern, with only 37% of the terrestrial OTUs significantly more represented in the terrestrial environment and 63% of the OTUs in the canopy environment. This difference between ectomycorrhizal and non-ectomycorrhizal patterns could be explained by the relative influence of the edaphic environment on ectomycorrhizal and non-ectomycorrhizal fungi. The supply of carbon to ectomycorrhizal fungi by the host roots means that those fungal species may have less reliance on the soil for this important element, whereas the non-ectomycorrhizal species (that span soil saprophytes, insect-associated fungi, parasites, and many other guilds) could be much more influenced by the soil environment, either directly because of carbon availability, or indirectly via the soil being host to other organisms. It is still possible that the ectomycorrhizal communities are too influenced by the soil organic matter. It is notable that the ectomycorrhizal communities in both canopy and terrestrial environments included many species of *Cortinarius*, with 15 out of the 25 most abundant OTUs in the canopy samples belonging to that genus, and the most abundant terrestrial OTU. *Cortinarius* may have a role in degradation of organic matter in soils due to the possession of class II peroxidases that degrade lignin [45]. The high organic matter content of the canopy soils may be driving ectomycorrhizal species assemblages in that environment by providing a substrate better exploited by fungi that can take advantage of it.

It is acknowledged that the non-mycorrhizal fungi in the hyphal ingrowth bags are a small and unusual subsample of the true diversity of non-ectomycorrhizal soil fungi, because we did not directly sample these fungi from the soil, but rather indirectly from the hyphal ingrowth bags. However, given that the hyphal ingrowth bags were uniform in the canopy and terrestrial sites, the patterns seen here for both ectomycorrhizal and non-ectomycorrhizal fungi do reflect the different source populations of fungi in either environment, and for that reason the patterns observed for both groups of fungi do have biological and meaningful relevance. Diversity of non-ectomycorrhizal fungi in Australian native mixed forest was compared with adjacent *Araucaria* plantation forest in Australia [46], measuring diversity using both total DNA extracted from soil and from hyphal ingrowth bags. The total soil fungal communities were found to be more dissimilar between treatments than the communities sampled from hyphal ingrowth bags, indicating that the hyphal ingrowth bags did accumulate particular groups of fungi. The fact we retrieved distinctly different non-ectomycorrhizal communities from hyphal ingrowth bags in the present study indicates that the source populations of fungi in the canopy and terrestrial environments are distinctly different. The large difference in organic matter between canopy and terrestrial environments, and the presence of canopy epiphytes, are likely factors affecting the different communities of fungi.

Twenty-two percent of the sequence reads recovered from the hyphal ingrowth bags (representing 71% of the OTUs) were not assigned to any functional group. Some of these reads were determined to be non-fungal or of very low identity to any sequence on GenBank, and thus were difficult to deal with in any systematic way. Further, it is possible that at least some of these are erroneous sequences generated by PCR and sequencing errors, and future work could involve the identification and exclusion of these [47]. In previous studies using cloning of DNA amplified from hyphal ingrowth bags, higher proportions of OTUs were found to belong to ectomycorrhizal fungi. For example, at least 88% of clones from hyphal ingrowth bags buried in soil in Australian *Eucalyptus pilularis* forest were from ectomycorrhizal families [17], whereas in the present study, only 9% of total OTUs and 57% of total reads could be assigned to ectomycorrhizal taxa. Potential explanations for the lower proportion of ectomycorrhizal reads in the present study are many and could be related to inherent differences in fungal communities associated with the different tree species at each site, or more likely differences in the sequencing methodologies used. The Illumina sequencing used in the present study detected 294,500 sequences, compared with 800 clones analysed in the *Eucalyptus* study, so this 370-fold increase in sequences has likely detected many more rare taxa, an acknowledged feature of next-generation sequencing studies [48]. Thus, sequencing errors and increased detection of rare and poorly known OTUs are likely contributors to the lower proportion of ectomycorrhizal fungi in hyphal ingrowth bags in the present study.

That canopy soils are rich in ectomycorrhizal fungi accords with our earlier root tip-survey in canopy soils at the same locality and with the same host tree species [10]. We did not quantify the proportion of ectomycorrhizal root tips or total mycelial biomass in the canopy versus terrestrial soils at this site, so it is not possible to address the absolute contribution of each habitat to the overall ectomycorrhizal community associated with these trees. In a Costa Rican tropical montane rainforest [49], living fine adventitious roots in the canopy of *Quercus copeyensis* trees comprised only < 0.04% of the biomass of living fine terrestrial roots, and were thus regarded as having a negligible contribution to the total fine root biomass of the stand of trees. Notably though, the *Q*. *copeyensis* canopy roots were not ectomycorrhizal, in contrast to the heavily colonized terrestrial roots, and thus the canopy roots may lack the support of ectomycorrhizal fungi to exploit the canopy soil habitat. The proportional biomass of canopy soils worldwide is thought to be relatively low [1], but a New Zealand study [2] close to the site of the present study found high biomass associated with another forest tree, *D*. *dacrydioides*, however this was not quantified for the *Nothofagus* trees in the present study. The canopy soil of *N*. *menziesii* does host a wide range of ectomycorrhizal species and should not be discounted in terms of its contribution to the richness of ectomycorrhizal fungi associated with these trees. In considering the ecosystem services the canopy habitat provides to the ectomycorrhizal fungal community, the canopy communities may act as a reservoir for ectomycorrhizal species, from which the terrestrial communities recruit as canopy individuals fruit or are dispersed vegetatively. Recruitment to the canopy from the terrestrial habitat is also possible, and the differential representation of many OTUs shown here indicates that (i) beta diversity is increased by the existence of the canopy community, and (ii) that there may be limitations to recruitment of some species from one habitat to the other. In the present study, it was difficult to control for stochastic processes with only five trees sampled, and a multi-site study to determine how generally applicable these phenomena are would be of value. The identification of ectomycorrhizal OTUs as largely indigenous (endemic or Australasian) was typical of the biogeography of the region [50], with strong affinities with NZ, and representatives from Australia, South America and some biological invaders from the Northern Hemisphere. Of potential biosecurity importance to New Zealand was the discovery of *Hebeloma hiemale* in the hyphal ingrowth bags. This species has been reported from New Zealand previously (including PDD88816, GenBank accession GQ86951 from *Salix caprea* L. [41] and OTA60226, GenBank accession JX178629 from under introduced *Quercus* sp. at Oakune, New Zealand, erroneously identified as *H*. *sacchariolens* in Teasdale *et al*. [51]). *Hebeloma hiemale* has a wide host range, including conifers and angiosperms [41]. The discovery here of *H*. *hiemale* in hyphal ingrowth bags from *Nothofagus*-associated soil indicates *H*. *hiemale* is a potential symbiont with this New Zealand native tree.

Ectomycorrhizal roots and fungi slow the soil carbon cycle, through competition with decomposers for nitrogen [52]. In an old-growth forest like that in the present study, where canopy soil accumulation is extensive, decreased decomposition in the canopy soil of trees where the adventitious canopy roots are ectomycorrhizal may contribute positively to the above-ground forest carbon budget, more so than the canopy soil of non-ectomycorrhizal host trees. How auto- and heterotrophic nitrogen-fixing bacteria contribute to canopy soil nitrogen, and how this relates to fungal diversity, organic matter accumulation and decomposition, may improve our understanding of carbon and nutrient dynamics in these forests.

## Supporting information

S1 Data List of OTUs and ITS sequences in fasta format. (FA)

S2 Data Table of read abundance OTU taxonomic identification and functional guild for each OTU in each sample. (XLSX)

S1 Fig Bayesian inference phylogeny of *Hebeloma*, indicating the phylogenetic position of OTU112. (PDF)

S1 Table Locations of the trees sampled in this study, hyphal ingrowth bag identifiers and height above ground level of each hyphal ingrowth bag buried in the canopy environment. All bags were buried at a depth of 3–4 cm.
(DOCX)

S2 Table Results of a PERMANOVA test (999 permutations) to determine if the centroids of canopy and terrestrial ectomycorrhizal communities (“Soil Type”) are significantly different. (DOCX)

S3 Table Results of a PERMANOVA test (999 permutations) to determine if the dispersion of canopy and terrestrial ectomycorrhizal communities are significantly different. (DOCX)

S4 Table Results of a PERMANOVA test (999 permutations) to determine if the centroids of canopy and terrestrial non-ectomycorrhizal communities (“Soil Type”) are significantly different. (DOCX)

S5 Table Results of a PERMANOVA test (999 permutations) to determine if the dispersion of canopy and terrestrial non-ectomycorrhizal communities are significantly different. (DOCX)

S6 Table Differential representation of ectomycorrhizal OTUs in terrestrial and canopy samples indicated by the Kruskal-Wallis test where *p* ≤ 0.05, ranked by false discovery rate and Bonferroni *p* values. OTUs with significant differential representation with Bonferroni-adjusted *p* values in either environment are shaded grey. The environment where each OTU dominates is shaded yellow.
(DOCX)

S7 Table Differential representation of non-ectomycorrhizal OTUs in terrestrial and canopy samples indicated by the Kruskal-Wallis test where *p* ≤ 0.05, ranked by false discovery rate and Bonferroni *p* values. OTUs with significant differential representation with Bonferroni-adjusted *p* values in either environment are shaded grey. The environment where each OTU dominates is shaded yellow.
(DOCX)
