# Supplementary figures and images for: Fungal diversity in canopy soil of silver beech, Nothofagus menziesii (Nothofagaceae)
Source: PLoS One. 2020 Jan 24;15(1):e0227860. doi: 10.1371/journal.pone.0227860 (PMC6980614; doi:10.1371/journal.pone.0227860)

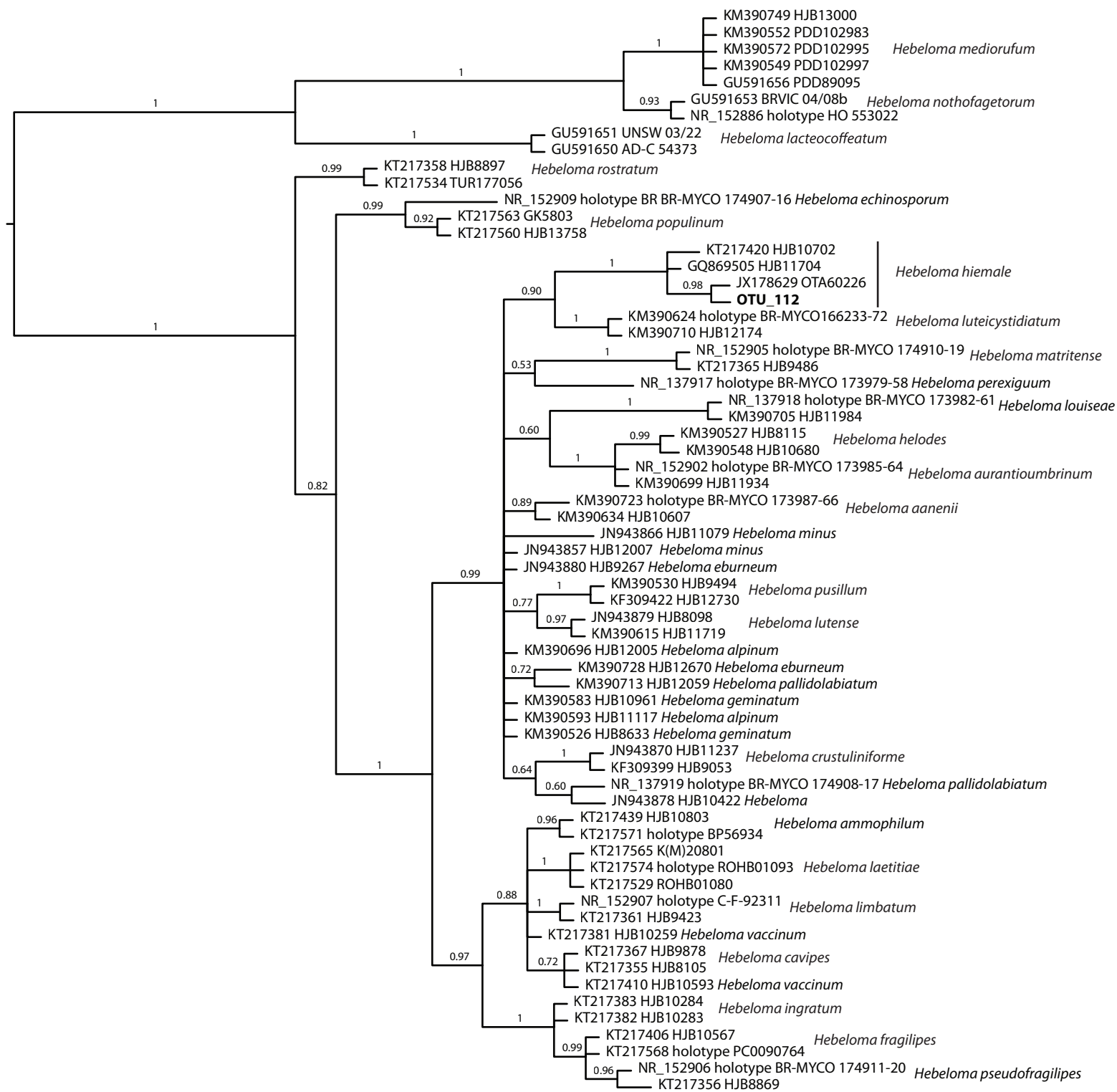

0.0080

Supplement: S1 Fig — (PDF) [file pone.0227860.s003.pdf]
