# Supplementary material for: Fungal diversity in canopy soil of silver beech, Nothofagus menziesii (Nothofagaceae)
Source: PLoS One. 2020 Jan 24;15(1):e0227860. doi: 10.1371/journal.pone.0227860 (PMC6980614; doi:10.1371/journal.pone.0227860)
Supplement: S1 Table — All bags were buried at a depth of 3–4 cm. (DOCX) [file pone.0227860.s004.docx]

**S1 Table** Locations of the trees sampled in this study, hyphal ingrowth bag identifiers and height above ground level of each hyphal ingrowth bag buried in the canopy environment. All bags were buried at a depth of 3–4 cm.

| **Tree** | **Location of tree (NZTM 2000 Map reference^a^)** | **Estimated tree height (m)** | **Approximate trunk diameter at breast height (m)** | **Soil environment** | **Initial bag number** | **Designated sample number** | **Height above ground of canopy samples (m)** |
| --- | --- | --- | --- | --- | --- | --- | --- |
| 1 | 1256287.69 E, 5113049.33 N | 14 | 1 | Canopy | 1 | 21 | 10.2 |
|  |  |  |  |  | 2^b^ |  | 9.5 |
|  |  |  |  |  | 3 | 22 | 8.0 |
|  |  |  |  |  | 4 | 23 | 11.5 |
|  |  |  |  |  | 5^b^ |  | 12.95 |
|  |  |  |  | Terrestrial | 6 | 1 |  |
|  |  |  |  |  | 7 | 2 |  |
|  |  |  |  |  | 8 | 3 |  |
|  |  |  |  |  | 9 | 4 |  |
|  |  |  |  |  | 10 | 5 |  |
| 2 | 1256340.09 E, 5113056.73 N | 19 | > 1.5 | Canopy | 11 | 24 | 17.3 |
|  |  |  |  |  | 12 | 25 | 14.2 |
|  |  |  |  |  | 13 | 26 | 14.7 |
|  |  |  |  |  | 14^b^ |  | 11.4 |
|  |  |  |  |  | 15 | 27 | 5.2 |
|  |  |  |  | Terrestrial | 16 | 6 |  |
|  |  |  |  |  | 17 | 7 |  |
|  |  |  |  |  | 18 | 8 |  |
|  |  |  |  |  | 19 | 9 |  |
|  |  |  |  |  | 20 | 10 |  |
| 3 | 1256375.57 E, 5113027.75 N | 14 | 1 | Canopy | 21 | 28 | 4.2 |
|  |  |  |  |  | 22^b^ |  | 9.3 |
|  |  |  |  |  | 23^b^ |  | 5.1 |
|  |  |  |  |  | 24^b^ |  | 5.8 |
|  |  |  |  |  | 25 | 29 | 12.7 |
|  |  |  |  | Terrestrial | 26 | 11 |  |
|  |  |  |  |  | 27^b^ |  |  |
|  |  |  |  |  | 28 | 12 |  |
|  |  |  |  |  | 29 | 13 |  |
|  |  |  |  |  | 30^b^ |  |  |
| 4 | 1256298.62 E, 5113054.92 N | 11 | 1 | Canopy | 31 | 30 | 1.9 |
|  |  |  |  |  | 32^b^ |  | 7.6 |
|  |  |  |  |  | 33^b^ |  | 7.5 |
|  |  |  |  |  | 34^b^ |  | 11.4 |
|  |  |  |  |  | 35^b^ |  | 4.3 |
|  |  |  |  | Terrestrial | 36 | 14 |  |
|  |  |  |  |  | 37 | 15 |  |
|  |  |  |  |  | 38 | 16 |  |
|  |  |  |  |  | 39 | 17 |  |
|  |  |  |  |  | 40 | 18 |  |
| 5 | 1256381.34 E, 5112983.87 N | 12 | 1 | Canopy | 41 | 31 | 9 |
|  |  |  |  |  | 42 | 32 | 6 |
|  |  |  |  |  | 43^b^ |  | 7.5 |
|  |  |  |  |  | 44^b^ |  | 10.2 |
|  |  |  |  |  | 45 | 33 | 3.6 |
|  |  |  |  | Terrestrial | 46 | 19 |  |
|  |  |  |  |  | 47^b^ |  |  |
|  |  |  |  |  | 48 | 20 |  |
|  |  |  |  |  | 49^b^ |  |  |
|  |  |  |  |  | 50^b^ |  |  |

^a^ GPS locations used the New Zealand Geodetic Datum 2000 (NZGD2000). ^b^ indicates samples that were either not buried or missing at time of collection
