# Supplementary material for: Fungal diversity in canopy soil of silver beech, Nothofagus menziesii (Nothofagaceae)
Source: PLoS One. 2020 Jan 24;15(1):e0227860. doi: 10.1371/journal.pone.0227860 (PMC6980614; doi:10.1371/journal.pone.0227860)
Supplement: S2 Table — (DOCX) [file pone.0227860.s005.docx]

**S2 Table.** Results of a PERMANOVA test (999 permutations) to determine if the centroids of canopy and terrestrial ectomycorrhizal communities (“Soil Type”) are significantly different.

|  | **Df** | **Sums of Sqs** | **Mean Sqs** | **F. Model** | **R^2^** | **Pr (>F)** |
| --- | --- | --- | --- | --- | --- | --- |
| **Soil Type** | 1 | 1.4999 | 1.49991 | 4.327 | 0.12983 | 0.001 |
| **Residuals** | 29 | 10.0527 | 0.34664 |  | 0.87017 |  |
| **Total** | 30 | 11.5526 |  |  | 1.00000 |  |
