# Supplementary material for: Fungal diversity in canopy soil of silver beech, Nothofagus menziesii (Nothofagaceae)
Source: PLoS One. 2020 Jan 24;15(1):e0227860. doi: 10.1371/journal.pone.0227860 (PMC6980614; doi:10.1371/journal.pone.0227860)
Supplement: S5 Table — (DOCX) [file pone.0227860.s008.docx]

**S5 Table.** Results of a PERMANOVA test (999 permutations) to determine if the dispersion of canopy and terrestrial non-ectomycorrhizal communities are significantly different.

|  | **Df** | **Sum Sq** | **Mean Sq** | **F** | **N. Perm** | **Pr (>F)** |
| --- | --- | --- | --- | --- | --- | --- |
| **Groups** | 1 | 0.003271 | 0.0032709 | 0.6491 | 999 | 0.434 |
| **Residuals** | 29 | 0.146145 | 0.0050395 |  |  |  |
