# Supplementary material for: Fungal diversity in canopy soil of silver beech, Nothofagus menziesii (Nothofagaceae)
Source: PLoS One. 2020 Jan 24;15(1):e0227860. doi: 10.1371/journal.pone.0227860 (PMC6980614; doi:10.1371/journal.pone.0227860)
Supplement: S6 Table — OTUs with significant differential representation with Bonferroni-adjusted p values in either environment are shaded grey. The environment where each OTU dominates is shaded yellow. (DOCX) [file pone.0227860.s009.docx]

**S6 Table** Differential representation of ectomycorrhizal OTUs in terrestrial and canopy samples indicated by the Kruskal-Wallis test where *p* ≤ 0.05, ranked by false discovery rate and Bonferroni *p* values. OTUs with significant differential representation with Bonferroni-adjusted *p* values in either environment are shaded grey. The environment where each OTU dominates is shaded yellow.

| **OTU** | **Test-Statistic** | **P** | **FDR P** | **Bonferroni P** | **Terrestrial mean** | **Canopy mean** | **Reference** | **Most similar species hypothesis (SH)** | **Species** | **Score** | **E-value** | **Percent** | **Taxonomy** |
| --- | --- | --- | --- | --- | --- | --- | --- | --- | --- | --- | --- | --- | --- |
| **OTU49** | 19.6718 | 0.0000 | 0.0022 | 0.0037 | 39.4 | 0.4 | [KP191861](https://unite.ut.ee/bl_forw.php?id=502694) | [SH2147768.08FU](https://unite.ut.ee/sh/SH2147768.08FU) | *Descomyces* sp. | 407 | 2.00E-112 | 100 | *Descomyces* sp. |
| **OTU387** | 18.3386 | 0.0000 | 0.0022 | 0.0074 | 108.3 | 0.9 | [GU222307](https://unite.ut.ee/bl_forw.php?id=15124) | [SH1546157.08FU](https://unite.ut.ee/sh/SH1546157.08FU#fndtn-panel1) | *Clavulina* sp. | 424 | 2.00E-117 | 98.33 | *Clavulina* sp. |
| **OTU234** | 17.8746 | 0.0000 | 0.0022 | 0.0094 | 23.7 | 0.5 | [KU685715](https://unite.ut.ee/bl_forw.php?id=758489) | [SH1553148.08FU](https://unite.ut.ee/sh/SH1553148.08FU#fndtn-panel1) | *Laccaria ohiensis* | 366.00 | 0.00 | 98.54 | *Laccaria* sp. |
| **OTU29** | 17.4999 | 0.0000 | 0.0022 | 0.0115 | 54.3 | 0.3 | [UDB014331](https://unite.ut.ee/bl_forw.php?id=382879) | [SH1504007.08FU](https://unite.ut.ee/sh/SH1504007.08FU#fndtn-panel1) | Envir: Cortinariaceae | 335 | 1.00E-90 | 96.12 | *Cortinarius* sp. |
| **OTU7** | 17.3316 | 0.0000 | 0.0022 | 0.0125 | 565.5 | 1.4 | [UDB002698](https://unite.ut.ee/bl_forw.php?id=3855) |  | Envir: Cantharellaceae | 283 | 4.00E-75 | 94.65 | Cantharellaceae sp. |
| **OTU2** | 17.2400 | 0.0000 | 0.0022 | 0.0131 | 834.7 | 0.6 | [JQ287673](https://unite.ut.ee/bl_forw.php?id=319812) | [SH2124709.08FU](https://unite.ut.ee/sh/SH2124709.08FU) | *Cortinarius thaumastus* | 335 | 8.00E-91 | 100 | *Cortinarius thaumastus* |
| **OTU76** | 16.4590 | 0.0000 | 0.0028 | 0.0198 | 32.5 | 0.2 | [GU980651](https://unite.ut.ee/bl_forw.php?id=201054) | [SH1527237.08FU](https://unite.ut.ee/sh/SH1527237.08FU#fndtn-panel1) | *Inocybe arthrocystis* | 147.00 | 0.00 | 83.63 | *Inocybe* sp. |
| **OTU16** | 15.6988 | 0.0001 | 0.0037 | 0.0296 | 0.2 | 282.5 | [EF634088](https://unite.ut.ee/bl_forw.php?id=56719) | [SH1528436.08FU](https://unite.ut.ee/sh/SH1528436.08FU#fndtn-panel1) | Thelephoraceae | 399 | 4.00E-110 | 100 | Thelephoraceae sp. |
| **OTU4470** | 14.8868 | 0.0001 | 0.0051 | 0.0456 | 409.5 | 0.0 | [JX648601](https://unite.ut.ee/bl_forw.php?id=381617) | [SH1504088.08FU](https://unite.ut.ee/sh/SH1504088.08FU#fndtn-panel1) | *Cortinarius* sp. | 322 | 7.00E-87 | 98.9 | *Cortinarius thaumastus* |
| **OTU105** | 14.0382 | 0.0002 | 0.0067 | 0.0715 | 35.4 | 0.5 | [KY827284](https://unite.ut.ee/bl_forw.php?id=862618) | [SH1562234.08FU](https://unite.ut.ee/sh/SH1562234.08FU#fndtn-panel1) | *Inocybe* sp. | 366.00 | 0.00 | 99.02 | *Inocybe* sp. |
| **OTU190** | 13.9731 | 0.0002 | 0.0067 | 0.0740 | 6.3 | 0.2 | [JQ408769](https://unite.ut.ee/bl_forw.php?id=347171) | [SH1641543.08FU](https://unite.ut.ee/sh/SH1641543.08FU#fndtn-panel1) | *Inocybe* sp. | 340.00 | 0.00 | 98.45 | *Inocybe* sp. |
| **OTU48** | 13.8253 | 0.0002 | 0.0067 | 0.0800 | 44.6 | 0.3 | [MF461604](https://unite.ut.ee/bl_forw.php?id=838622) | [SH2310360.08FU](https://unite.ut.ee/sh/SH2310360.08FU) | *Russula griseobrunnea* | 418 | 1.00E-115 | 100 | *Russula* sp. |
| **OTU747** | 13.2567 | 0.0003 | 0.0075 | 0.1084 | 7.4 | 0.2 | [KU685711](https://unite.ut.ee/bl_forw.php?id=758493) | [SH1553003.08FU](https://unite.ut.ee/sh/SH1553003.08FU#fndtn-panel1) | *Laccaria glabripes* | 357.00 | 0.00 | 98.05 | Hydnangiaceae sp. |
| **OTU26** | 13.1902 | 0.0003 | 0.0075 | 0.1123 | 55.3 | 0.1 | [KU523937](https://unite.ut.ee/bl_forw.php?id=796949) | [SH2147886.08FU](https://unite.ut.ee/sh/SH2147886.08FU) | *Descolea gunnii* | 387 | 3.00E-106 | 100 | *Descolea gunnii* |
| **OTU353** | 13.0920 | 0.0003 | 0.0075 | 0.1183 | 1.3 | 5.2 | [JX178630](https://unite.ut.ee/bl_forw.php?id=371155) | [SH1520338.08FU](https://unite.ut.ee/sh/SH1520338.08FU#fndtn-panel1) | *Tricholoma elegans* | 379 | 4.00E-104 | 100 | *Tricholoma elegans* |
| **OTU10** | 12.9968 | 0.0003 | 0.0075 | 0.1245 | 186.8 | 0.2 | [KY684373](https://unite.ut.ee/bl_forw.php?id=933868) | [SH1650399.08FU](https://unite.ut.ee/sh/SH1650399.08FU#fndtn-panel1) | Cantharellaceae sp. | 292 | 6.00E-78 | 96.11 | Cantharellaceae sp. |
| **OTU226** | 12.9486 | 0.0003 | 0.0075 | 0.1277 | 0.2 | 119.9 | [UDB004029](https://unite.ut.ee/bl_forw.php?id=4012) | [SH1528630.08FU](https://unite.ut.ee/sh/SH1528630.08FU#fndtn-panel1) | Envir: Thelephoraceae | 350 | 4.00E-95 | 95.43 | *Tomentella* sp. |
| **OTU61** | 12.4745 | 0.0004 | 0.0090 | 0.1646 | 6.1 | 36.0 | [KY462421](https://unite.ut.ee/bl_forw.php?id=732868) | [SH1504760.08FU](https://unite.ut.ee/sh/SH1504760.08FU#fndtn-panel1) | *Cortinarius* sp. | 257 | 2.00E-67 | 91.15 | *Cortinarius* sp. |
| **OTU55** | 12.2420 | 0.0005 | 0.0090 | 0.1864 | 12.4 | 88.6 | [MH101610](https://unite.ut.ee/bl_forw.php?id=909314) | [SH1504292.08FU](https://unite.ut.ee/sh/SH1504292.08FU#fndtn-panel1) | *Cortinarius cucumeris* | 361 | 2.00E-98 | 98.54 | *Cortinarius cucumeris* |
| **OTU134** | 12.2045 | 0.0005 | 0.0090 | 0.1902 | 3.7 | 14.5 | [MH101581](https://unite.ut.ee/bl_forw.php?id=909343) | [SH2122340.08FU](https://unite.ut.ee/sh/SH2122340.08FU) | *Cortinarius* sp. | 368 | 9.00E-101 | 100 | *Cortinarius* sp. |
| **OTU2227** | 12.1697 | 0.0005 | 0.0090 | 0.1938 | 3.0 | 0.0 | [KU685711](https://unite.ut.ee/bl_forw.php?id=758493) | [SH1553003.08FU](https://unite.ut.ee/sh/SH1553003.08FU#fndtn-panel1) | *Laccaria glabripes* | 357.00 | 0.00 | 98.05 | *Laccaria* sp. |
| **OTU3083** | 12.1226 | 0.0005 | 0.0090 | 0.1988 | 51.0 | 0.0 | [GU222307](https://unite.ut.ee/bl_forw.php?id=15124) | [SH1546157.08FU](https://unite.ut.ee/sh/SH1546157.08FU#fndtn-panel1) | *Clavulina* sp. | 401 | 1.00E-110 | 96.67 | *Clavulina* sp. |
| **OTU80** | 11.9034 | 0.0006 | 0.0092 | 0.2236 | 24.0 | 77.1 | [KT334128](https://unite.ut.ee/bl_forw.php?id=711954) | [SH2123685.08FU](https://unite.ut.ee/sh/SH2123685.08FU) | *Cortinarius porphyroideus* | 372 | 7.00E-102 | 100 | Cortinariaceae sp. |
| **OTU69** | 11.8975 | 0.0006 | 0.0092 | 0.2243 | 18.1 | 0.1 | [KF819809](https://unite.ut.ee/bl_forw.php?id=419533) | [SH1558615.08FU](https://unite.ut.ee/sh/SH1558615.08FU#fndtn-panel1) | *Cystangium nothofagi* | 431.00 | 0.00 | 95.54 | *Russula* sp. |
| **OTU59** | 11.8453 | 0.0006 | 0.0092 | 0.2306 | 19.2 | 83.5 | [MG552976](https://unite.ut.ee/bl_forw.php?id=940911) | [SH2122659.08FU](https://unite.ut.ee/sh/SH2122659.08FU) | *Cortinarius* sp. | 361 | 2.00E-98 | 99.5 | *Cortinarius* sp. |
| **OTU3826** | 11.6335 | 0.0006 | 0.0099 | 0.2585 | 27.4 | 0.1 | [KU685706](https://unite.ut.ee/bl_forw.php?id=758498) | [SH1553106.08FU](https://unite.ut.ee/sh/SH1553106.08FU#fndtn-panel1) | *Laccaria fibrillosa* | 370.00 | 0.00 | 99.02 | Hydnangiaceae sp. |
| **OTU327** | 11.4314 | 0.0007 | 0.0107 | 0.2881 | 0.4 | 7.9 | [GU233331](https://unite.ut.ee/bl_forw.php?id=5021) | [SH1504704.08FU](https://unite.ut.ee/sh/SH1504704.08FU#fndtn-panel1) | *Cortinarius porphyrophaeus* | 372.00 | 0.00 | 100.00 | *Cortinarius porphyrophaeus* |
| **OTU56** | 11.3033 | 0.0008 | 0.0110 | 0.3087 | 7.1 | 49.8 | [KJ635239](https://unite.ut.ee/bl_forw.php?id=473309) | [SH2122019.08FU](https://unite.ut.ee/sh/SH2122019.08FU) | *Cortinarius veronicae* | 372 | 7.00E-102 | 100 | *Cortinarius veronicoides* |
| **OTU147** | 11.2283 | 0.0008 | 0.0111 | 0.3214 | 5.9 | 20.9 | [KC017360](https://unite.ut.ee/bl_forw.php?id=378228) | [SH2121746.08FU](https://unite.ut.ee/sh/SH2121746.08FU) | *Cortinarius* sp. | 368 | 9.00E-101 | 99.5 | *Dermocybe cardinalis* |
| **OTU158** | 10.9951 | 0.0009 | 0.0121 | 0.3645 | 3.2 | 7.5 | [MH270626](https://unite.ut.ee/bl_forw.php?id=894332) | [SH1545229.08FU](https://unite.ut.ee/sh/SH1545229.08FU#fndtn-panel1) | *Cortinarius hemitrichus* | 342.00 | 0.00 | 100.00 | *Cortinarius comptulus* |
| **OTU24** | 10.8202 | 0.0010 | 0.0126 | 0.4006 | 85.9 | 1.0 | [MH019833](https://unite.ut.ee/bl_forw.php?id=913163) | [SH1551663.08FU](https://unite.ut.ee/sh/SH1551663.08FU#fndtn-panel1) | Fungi | 294 | 2.00E-78 | 89.17 | Clavulinaceae sp. |
| **OTU18** | 10.8085 | 0.0010 | 0.0126 | 0.4031 | 81.5 | 0.8 | [UDB014880](https://unite.ut.ee/bl_forw.php?id=596678) |  | Envir: Pezizales | 318 | 8.00E-86 | 98.88 | *Tarzetta* sp. |
| **OTU83** | 10.7199 | 0.0011 | 0.0128 | 0.4229 | 10.5 | 28.6 | [JQ282169](https://unite.ut.ee/bl_forw.php?id=349588) | [SH1504725.08FU](https://unite.ut.ee/sh/SH1504725.08FU#fndtn-panel1) | *Cortinarius* sp. | 333 | 3.00E-90 | 97 | *Cortinarius* sp. |
| **OTU35** | 10.6457 | 0.0011 | 0.0129 | 0.4402 | 79.3 | 0.7 | [KY462407](https://unite.ut.ee/bl_forw.php?id=732882) | [SH1651300.08FU](https://unite.ut.ee/sh/SH1651300.08FU#fndtn-panel1) | *Inocybe* sp. | 169 | 1.00E-40 | 90.3 | *Inocybe* sp. |
| **OTU357** | 10.5357 | 0.0012 | 0.0129 | 0.4672 | 0.3 | 4.1 | [MH101526](https://unite.ut.ee/bl_forw.php?id=909398) | [SH1504532.08FU](https://unite.ut.ee/sh/SH1504532.08FU#fndtn-panel1) | *Cortinarius* sp. | 374.00 | 0.00 | 99.51 | *Cortinarius* sp. |
| **OTU64** | 10.5087 | 0.0012 | 0.0129 | 0.4741 | 23.0 | 0.6 | [KP308755](https://unite.ut.ee/bl_forw.php?id=514354) | [SH1568966.08FU](https://unite.ut.ee/sh/SH1568966.08FU#fndtn-panel1) | *Inocybe leptospermi* | 267.00 | 0.00 | 91.75 | *Inocybe* sp. |
| **OTU27** | 10.4932 | 0.0012 | 0.0129 | 0.4781 | 45.9 | 137.8 | [MG019344](https://unite.ut.ee/bl_forw.php?id=955676) | [SH2122097.08FU](https://unite.ut.ee/sh/SH2122097.08FU) | *Cortinarius* sp. | 361 | 2.00E-98 | 99 | *Cortinarius* sp. |
| **OTU186** | 10.3915 | 0.0013 | 0.0133 | 0.5051 | 0.0 | 8.9 | [MH101541](https://unite.ut.ee/bl_forw.php?id=909383) | [SH1504240.08FU](https://unite.ut.ee/sh/SH1504240.08FU#fndtn-panel1) | *Cortinarius* sp. | 363.00 | 0.00 | 99.50 | *Cortinarius* sp. |
| **OTU138** | 10.2229 | 0.0014 | 0.0140 | 0.5535 | 3.2 | 11.1 | [JX178624](https://unite.ut.ee/bl_forw.php?id=371158) | [SH2137786.08FU](https://unite.ut.ee/sh/SH2137786.08FU) | *Inocybe calamistratoides* | 390 | 2.00E-107 | 100 | *Inocybe calamistratoides* |
| **OTU6** | 10.1953 | 0.0014 | 0.0140 | 0.5618 | 386.1 | 0.9 | [UDB004029](https://unite.ut.ee/bl_forw.php?id=4012) | [SH1528630.08FU](https://unite.ut.ee/sh/SH1528630.08FU#fndtn-panel1) | Envir: Thelephoraceae | 359 | 6.00E-98 | 96.35 | *Tomentella* sp. |
| **OTU53** | 9.8318 | 0.0017 | 0.0167 | 0.6844 | 26.5 | 0.2 | [KJ547666](https://unite.ut.ee/bl_forw.php?id=458045) | [SH1504788.08FU](https://unite.ut.ee/sh/SH1504788.08FU#fndtn-panel1) | *Cortinarius aurantioferreus* | 363.00 | 0.00 | 99.01 | Cortinariaceae sp. |
| **OTU452** | 9.6712 | 0.0019 | 0.0176 | 0.7468 | 2.6 | 0.0 | [MH019880](https://unite.ut.ee/bl_forw.php?id=913116) | [SH1503259.08FU](https://unite.ut.ee/sh/SH1503259.08FU#fndtn-panel1) | Fungi | 377 | 2.00E-103 | 97.72 | Thelephoraceae sp. |
| **OTU75** | 9.6494 | 0.0019 | 0.0176 | 0.7558 | 16.7 | 42.5 | [LT000117](https://unite.ut.ee/bl_forw.php?id=535791) | [SH1647807.08FU](https://unite.ut.ee/sh/SH1647807.08FU#fndtn-panel1) | *Tricholoma viridiolivaceum* | 372 | 7.00E-102 | 100 | *Tricholoma viridiolivaceum* |
| **OTU136** | 9.5424 | 0.0020 | 0.0178 | 0.8011 | 5.6 | 22.5 | [MH101523](https://unite.ut.ee/bl_forw.php?id=909401) | [SH2586004.08FU](https://unite.ut.ee/sh/SH2586004.08FU) | *Cortinarius* sp. | 248 | 8.00E-65 | 100 | *Cortinarius* sp. |
| **OTU225** | 9.5332 | 0.0020 | 0.0178 | 0.8051 | 20.9 | 0.6 | [KU685753](https://unite.ut.ee/bl_forw.php?id=758451) | [SH1553110.08FU](https://unite.ut.ee/sh/SH1553110.08FU#fndtn-panel1) | *Laccaria* sp. | 381.00 | 0.00 | 97.71 | *Laccaria* sp. |
| **OTU37** | 9.5027 | 0.0021 | 0.0178 | 0.8186 | 20.1 | 82.8 | [KP191825](https://unite.ut.ee/bl_forw.php?id=502730) | [SH2288501.08FU](https://unite.ut.ee/sh/SH2288501.08FU) | *Austropaxillus macnabbii* | 387 | 3.00E-106 | 100 | *Austropaxillus* sp. |
| **OTU20** | 9.2081 | 0.0024 | 0.0205 | 0.9614 | 79.1 | 1.0 | [KP636873](https://unite.ut.ee/bl_forw.php?id=515287) | [SH1562206.08FU](https://unite.ut.ee/sh/SH1562206.08FU#fndtn-panel1) | *Astrosporina subclavata* | 318 | 9.00E-86 | 97.85 | Inocybaceae sp. |
| **OTU126** | 9.1557 | 0.0025 | 0.0206 | 0.9893 | 8.2 | 32.6 | [MH101550](https://unite.ut.ee/bl_forw.php?id=909374) | [SH2123955.08FU](https://unite.ut.ee/sh/SH2123955.08FU) | *Cortinarius rotundisporus* | 374 | 2.00E-102 | 100 | *Cortinarius* sp. |
| **OTU293** | 9.0531 | 0.0026 | 0.0214 | 1.0000 | 0.9 | 3.7 | [JX000356](https://unite.ut.ee/bl_forw.php?id=306783) | [SH1506024.08FU](https://unite.ut.ee/sh/SH1506024.08FU#fndtn-panel1) | *Cortinarius luteinus* | 363.00 | 0.00 | 100.00 | *Cortinarius* sp. |
| **OTU230** | 8.8551 | 0.0029 | 0.0233 | 1.0000 | 1.4 | 6.2 | [KU612629](https://unite.ut.ee/bl_forw.php?id=699508) | [SH1568886.08FU](https://unite.ut.ee/sh/SH1568886.08FU#fndtn-panel1) | *Hydnum* sp. | 416.00 | 0.00 | 98.33 | *Hydnum* sp. |
| **OTU14** | 8.7861 | 0.0030 | 0.0237 | 1.0000 | 221.5 | 0.8 | [KF871770](https://unite.ut.ee/bl_forw.php?id=474226) | [SH1562311.08FU](https://unite.ut.ee/sh/SH1562311.08FU#fndtn-panel1) | *Inocybe* sp. | 337 | 3.00E-91 | 93.86 | *Inocybe* sp. |
| **OTU949** | 8.5946 | 0.0034 | 0.0259 | 1.0000 | 1.1 | 3.6 | [UDB014328](https://unite.ut.ee/bl_forw.php?id=382876) | [SH1503826.08FU](https://unite.ut.ee/sh/SH1503826.08FU#fndtn-panel1) | Envir: Cortinariaceae | 326.00 | 0.00 | 96.92 | Cortinariaceae sp. |
| **OTU200** | 8.2022 | 0.0042 | 0.0315 | 1.0000 | 3.2 | 10.9 | [MH101637](https://unite.ut.ee/bl_forw.php?id=909287) | [SH1503872.08FU](https://unite.ut.ee/sh/SH1503872.08FU#fndtn-panel1) | *Cortinarius elaiochrous* | 353.00 | 0.00 | 98.98 | *Cortinarius elaiochrous* |
| **OTU1781** | 8.0376 | 0.0046 | 0.0329 | 1.0000 | 0.0 | 1.2 | [KJ635239](https://unite.ut.ee/bl_forw.php?id=473309) | [SH1503791.08FU](https://unite.ut.ee/sh/SH1503791.08FU#fndtn-panel1) | *Cortinarius veronicae* | 307.00 | 0.00 | 94.53 | *Cortinarius veronicoides* |
| **OTU41** | 8.0328 | 0.0046 | 0.0329 | 1.0000 | 0.0 | 66.4 | [KJ635245](https://unite.ut.ee/bl_forw.php?id=473303) | [SH1503938.08FU](https://unite.ut.ee/sh/SH1503938.08FU#fndtn-panel1) | *Cortinarius orixanthus* | 320 | 3.00E-86 | 95.1 | *Cortinarius* sp. |
| **OTU106** | 8.0163 | 0.0046 | 0.0329 | 1.0000 | 26.5 | 0.1 | [KP171136](https://unite.ut.ee/bl_forw.php?id=523861) | [SH1542785.08FU](https://unite.ut.ee/sh/SH1542785.08FU#fndtn-panel1) | *Inocybe* sp. | 206.00 | 0.00 | 92.00 | *Inocybe* sp. |
| **OTU34** | 7.9888 | 0.0047 | 0.0329 | 1.0000 | 34.4 | 0.4 | [KP641632](https://unite.ut.ee/bl_forw.php?id=515267) | [SH1568962.08FU](https://unite.ut.ee/sh/SH1568962.08FU#fndtn-panel1) | *Inocybe* sp. | 351.00 | 0.00 | 99.48 | *Inocybe* sp. |
| **OTU40** | 7.7822 | 0.0053 | 0.0363 | 1.0000 | 62.7 | 0.5 | [GU222324](https://unite.ut.ee/bl_forw.php?id=15107) | [SH2272056.08FU](https://unite.ut.ee/sh/SH2272056.08FU) | *Russula roseostipitata* | 503 | 3.00E-141 | 100 | *Russula roseostipitata* |
| **OTU112** | 7.6368 | 0.0057 | 0.0387 | 1.0000 | 1.9 | 19.1 | [JX178629](https://unite.ut.ee/bl_forw.php?id=370848) | [SH2291742.08FU](https://unite.ut.ee/sh/SH2291742.08FU) | *Hebeloma hiemale* | 398 | 1.00E-109 | 100 | *Hebeloma hiemale* |
| **OTU150** | 7.5044 | 0.0062 | 0.0393 | 1.0000 | 6.6 | 0.0 | [GU233360](https://unite.ut.ee/bl_forw.php?id=4992) | [SH1562212.08FU](https://unite.ut.ee/sh/SH1562212.08FU#fndtn-panel1) | *Astrosporina subclavata* | 339.00 | 0.00 | 100.00 | Inocybaceae sp. |
| **OTU257** | 7.5022 | 0.0062 | 0.0393 | 1.0000 | 2.3 | 0.0 | [JF960838](https://unite.ut.ee/bl_forw.php?id=227594) | [SH1502883.08FU](https://unite.ut.ee/sh/SH1502883.08FU#fndtn-panel1) | Thelephoraceae | 333 | 4.00E-90 | 94.86 | Thelephoraceae sp. |
| **OTU305** | 7.4956 | 0.0062 | 0.0393 | 1.0000 | 2.5 | 0.0 | [JX000372](https://unite.ut.ee/bl_forw.php?id=306775) | [SH1504804.08FU](https://unite.ut.ee/sh/SH1504804.08FU#fndtn-panel1) | *Cortinarius pectochelis* | 372.00 | 0.00 | 100.00 | *Cortinarius pectochelis* |
| **OTU221** | 7.4890 | 0.0062 | 0.0393 | 1.0000 | 4.3 | 0.0 | [JF960838](https://unite.ut.ee/bl_forw.php?id=227594) | [SH1502883.08FU](https://unite.ut.ee/sh/SH1502883.08FU#fndtn-panel1) | Thelephoraceae | 339 | 8.00E-92 | 95.33 | Thelephoraceae sp. |
| **OTU217** | 7.3100 | 0.0069 | 0.0427 | 1.0000 | 0.7 | 5.8 | [MG552975](https://unite.ut.ee/bl_forw.php?id=940912) | [SH1503760.08FU](https://unite.ut.ee/sh/SH1503760.08FU#fndtn-panel1) | *Cortinarius* sp. | 368.00 | 0.00 | 100.00 | Cortinariaceae sp. |
| **OTU12** | 7.2372 | 0.0071 | 0.0438 | 1.0000 | 195.9 | 2.0 | [GU222261](https://unite.ut.ee/bl_forw.php?id=15170) | [SH2272053.08FU](https://unite.ut.ee/sh/SH2272053.08FU) | *Russula tricholomopsis* | 501 | 1.00E-140 | 99.64 | *Russula tricholomopsis* |
| **OTU148** | 7.0064 | 0.0081 | 0.0491 | 1.0000 | 1.3 | 13.1 | [MG019349](https://unite.ut.ee/bl_forw.php?id=955671) | [SH2578854.08FU](https://unite.ut.ee/sh/SH2578854.08FU) | *Cortinarius vitreopileatus* | 357 | 2.00E-97 | 100 | *Cortinarius vitreopileatus* |
