# Supplementary material for: Fungal diversity in canopy soil of silver beech, Nothofagus menziesii (Nothofagaceae)
Source: PLoS One. 2020 Jan 24;15(1):e0227860. doi: 10.1371/journal.pone.0227860 (PMC6980614; doi:10.1371/journal.pone.0227860)
Supplement: S7 Table — OTUs with significant differential representation with Bonferroni-adjusted p values in either environment are shaded grey. The environment where each OTU dominates is shaded yellow. (DOCX) [file pone.0227860.s010.docx]

**S7 Table** Differential representation of non-ectomycorrhizal OTUs in terrestrial and canopy samples indicated by the Kruskal-Wallis test where *p* ≤ 0.05, ranked by false discovery rate and Bonferroni *p* values. OTUs with significant differential representation with Bonferroni-adjusted *p* values in either environment are shaded grey. The environment where each OTU dominates is shaded yellow.

| **OTU** | **Test-Statistic** | **P** | **FDR P** | **Bonferroni P** | **Terrestrial mean** | **Canopy mean** | **Reference** | **Most similar species hypothesis (SH)** | **Species** | **Score** | **E-value** | **Percent** | **Taxonomy** |
| --- | --- | --- | --- | --- | --- | --- | --- | --- | --- | --- | --- | --- | --- |
| **OTU144** | 20.7925 | 0.0000 | 0.0031 | 0.0047 | 14.3 | 0.4 | [KP311421](https://unite.ut.ee/bl_forw.php?id=587465) | [SH2267003.08FU](https://unite.ut.ee/sh/SH2267003.08FU) | *Mortierella* sp. | 460 | 2.00E-128 | 99.6 | *Mortierella gamsii* |
| **OTU15** | 20.2460 | 0.0000 | 0.0031 | 0.0063 | 1.8 | 328.6 | [MG020711](https://unite.ut.ee/bl_forw.php?id=955512) | [SH2268660.08FU](https://unite.ut.ee/sh/SH2268660.08FU) | *Naganishia albida* | 405 | 8.00E-112 | 100 | *Naganishia* sp. |
| **OTU265** | 19.2125 | 0.0000 | 0.0036 | 0.0108 | 0.8 | 19.4 | [MG915522](https://unite.ut.ee/bl_forw.php?id=923802) | - | Fungi | 257 | 2.00E-67 | 93.18 | *Exobasidium* sp. |
| **OTU411** | 15.9636 | 0.0001 | 0.0146 | 0.0595 | 0.1 | 6.6 | [KY073419](https://unite.ut.ee/bl_forw.php?id=759776) | [SH1529988.08FU](https://unite.ut.ee/sh/SH1529988.08FU#fndtn-panel1) | *Penicillium* sp. | 291 | 2.00E-77 | 100 | *Penicillium* sp. |
| **OTU196** | 15.5742 | 0.0001 | 0.0146 | 0.0731 | 0.0 | 13.9 | [KC009082](https://unite.ut.ee/bl_forw.php?id=397405) | [SH1522825.08FU](https://unite.ut.ee/sh/SH1522825.08FU#fndtn-panel1) | *Clonostachys rosea* | 305 | 6.00E-82 | 100 | Bionectriaceae sp. |
| **OTU11** | 14.7605 | 0.0001 | 0.0161 | 0.1124 | 178.4 | 19.6 | [MG052956](https://unite.ut.ee/bl_forw.php?id=915861) | [SH2444324.08FU](https://unite.ut.ee/sh/SH2444324.08FU) | *Mortierella humilis* | 440 | 2.00E-122 | 100 | *Mortierella humilis* |
| **OTU17** | 14.6347 | 0.0001 | 0.0161 | 0.1202 | 12.8 | 258.4 | [X93980](https://unite.ut.ee/bl_forw.php?id=138314) | [SH2303512.08FU](https://unite.ut.ee/sh/SH2303512.08FU) | *Trichoderma viride* | 324 | 2.00E-87 | 100 | *Trichoderma* sp. |
| **OTU5** | 14.2620 | 0.0002 | 0.0161 | 0.1465 | 0.8 | 1090.3 | [KX222388](https://unite.ut.ee/bl_forw.php?id=798827) | - | Fungi | 351 | 1.00E-95 | 94.71 | Ceratobasidiaceae sp. |
| **OTU129** | 14.0330 | 0.0002 | 0.0161 | 0.1654 | 12.8 | 0.8 | [EF029209](https://unite.ut.ee/bl_forw.php?id=69949) | [SH1523256.08FU](https://unite.ut.ee/sh/SH1523256.08FU#fndtn-panel1) | *Chalara dualis* | 254 | 2.00E-66 | 97.95 | *Chalara* sp. |
| **OTU270** | 13.9405 | 0.0002 | 0.0161 | 0.1738 | 0.2 | 6.3 | [MH161248](https://unite.ut.ee/bl_forw.php?id=904266) | [SH1529989.08FU](https://unite.ut.ee/sh/SH1529989.08FU#fndtn-panel1) | *Penicillium melinii* | 302 | 8.00E-81 | 100 | *Penicillium* sp. |
| **OTU96** | 13.7405 | 0.0002 | 0.0161 | 0.1933 | 13.2 | 0.2 | [JX975915](https://unite.ut.ee/bl_forw.php?id=384337) | [SH2269093.08FU](https://unite.ut.ee/sh/SH2269093.08FU) | *Mortierella globulifera* | 433 | 4.00E-120 | 100 | *Mortierella globulifera* |
| **OTU51** | 13.5652 | 0.0002 | 0.0161 | 0.2122 | 67.5 | 0.2 | [KX640357](https://unite.ut.ee/bl_forw.php?id=759640) | [SH2266986.08FU](https://unite.ut.ee/sh/SH2266986.08FU) | *Mortierella* sp. | 459 | 7.00E-128 | 100 | *Mortierella* sp. |
| **OTU44** | 13.4637 | 0.0002 | 0.0161 | 0.2240 | 43.2 | 0.0 | [KX222781](https://unite.ut.ee/bl_forw.php?id=798434) | - | Fungi | 486 | 3.00E-136 | 100 | *Mortierella sarnyensis* |
| **OTU127** | 13.4542 | 0.0002 | 0.0161 | 0.2251 | 8.7 | 0.0 | [KT290029](https://unite.ut.ee/bl_forw.php?id=583828) | [SH1610166.08FU](https://unite.ut.ee/sh/SH1610166.08FU#fndtn-panel1) | *Cylindrocladiella* sp. | 287 | 2.00E-76 | 98.17 | *Cylindrocladiella* sp. |
| **OTU263** | 13.2983 | 0.0003 | 0.0163 | 0.2447 | 0.2 | 15.1 | [JN206398](https://unite.ut.ee/bl_forw.php?id=390407) | [SH1522258.08FU](https://unite.ut.ee/sh/SH1522258.08FU#fndtn-panel1) | *Umbelopsis isabellina* | 355 | 7.00E-97 | 99.49 | *Umbelopsis isabellina* |
| **OTU72** | 13.0411 | 0.0003 | 0.0164 | 0.2807 | 4.5 | 36.5 | [GU559986](https://unite.ut.ee/bl_forw.php?id=166563) | [SH2444871.08FU](https://unite.ut.ee/sh/SH2444871.08FU) | *Mortierella fimbricystis* | 440 | 2.00E-122 | 100 | *Mortierella* sp. |
| **OTU881** | 12.9516 | 0.0003 | 0.0164 | 0.2944 | 0.0 | 1.5 | [MG190565](https://unite.ut.ee/bl_forw.php?id=951481) | [SH1522969.08FU](https://unite.ut.ee/sh/SH1522969.08FU#fndtn-panel1) | *Ascomycota* sp. | 272 | 5.00E-72 | 100 | Hyaloscyphaceae sp. |
| **OTU21** | 12.9417 | 0.0003 | 0.0164 | 0.2960 | 3.2 | 129.4 | [EU552153](https://unite.ut.ee/bl_forw.php?id=43298) | [SH1614513.08FU](https://unite.ut.ee/sh/SH1614513.08FU#fndtn-panel1) | *Pyrenochaeta inflorescentiae* | 291 | 2.00E-77 | 98.2 | *Pyrenochaeta* sp. |
| **OTU50** | 12.5436 | 0.0004 | 0.0193 | 0.3662 | 0.2 | 53.3 | [JX976121](https://unite.ut.ee/bl_forw.php?id=384131) | [SH2267026.08FU](https://unite.ut.ee/sh/SH2267026.08FU) | *Mortierella gemmifera* | 473 | 3.00E-132 | 100 | *Mortierella gemmifera* |
| **OTU81** | 12.2456 | 0.0005 | 0.0215 | 0.4295 | 0.5 | 25.7 | [MH753702](https://unite.ut.ee/bl_forw.php?id=856868) | [SH2272412.08FU](https://unite.ut.ee/sh/SH2272412.08FU) | *Rhodotorula diobovata* | 390 | 2.00E-107 | 100 | *Rhodotorula* sp. |
| **OTU60** | 12.1021 | 0.0005 | 0.0221 | 0.4639 | 28.7 | 0.0 | [JX270502](https://unite.ut.ee/bl_forw.php?id=371643) | [SH2480509.08FU](https://unite.ut.ee/sh/SH2480509.08FU) | *Apiotrichum* sp. | 303 | 2.00E-81 | 100 | *Apiotrichum* sp. |
| **OTU38** | 11.9467 | 0.0005 | 0.0224 | 0.5042 | 11.7 | 182.9 | [JX976028](https://unite.ut.ee/bl_forw.php?id=384224) | [SH2267512.08FU](https://unite.ut.ee/sh/SH2267512.08FU) | *Mortierella zonata* | 448 | 1.00E-124 | 99.59 | *Mortierella zonata* |
| **OTU324** | 11.9083 | 0.0006 | 0.0224 | 0.5147 | 2.6 | 0.1 | [JN104514](https://unite.ut.ee/bl_forw.php?id=259799) | - | *Mortierella* sp. | 405 | 9.00E-112 | 95.98 | *Mortierella* sp. |
| **OTU227** | 11.7518 | 0.0006 | 0.0233 | 0.5598 | 4.5 | 0.1 | [EF029206](https://unite.ut.ee/bl_forw.php?id=69952) | [SH1648741.08FU](https://unite.ut.ee/sh/SH1648741.08FU#fndtn-panel1) | *Dendrosporium* sp. | 267 | 3.00E-70 | 98.67 | *Dendrosporium* sp. |
| **OTU644** | 11.5702 | 0.0007 | 0.0247 | 0.6172 | 0.1 | 2.3 | [KX403911](https://unite.ut.ee/bl_forw.php?id=792924) | - | *Dothideomycetes* sp. | 228 | 1.00E-58 | 95.14 | *Venturia* sp. |
| **OTU47** | 10.9245 | 0.0009 | 0.0336 | 0.8740 | 62.1 | 5.5 | [KY558367](https://unite.ut.ee/bl_forw.php?id=795501) | [SH2574334.08FU](https://unite.ut.ee/sh/SH2574334.08FU) | *Solicoccozyma terricola* | 433 | 4.00E-120 | 100 | *Solicoccozyma terricola* |
| **OTU385** | 10.6935 | 0.0011 | 0.0367 | 0.9902 | 0.3 | 6.2 | [JN206382](https://unite.ut.ee/bl_forw.php?id=390423) | [SH1557750.08FU](https://unite.ut.ee/sh/SH1557750.08FU#fndtn-panel1) | *Umbelopsis ramanniana* | 396 | 5.00E-109 | 99.09 | *Umbelopsis* sp. |
| **OTU45** | 10.3864 | 0.0013 | 0.0403 | 1.0000 | 0.0 | 76.3 | [KU063815](https://unite.ut.ee/bl_forw.php?id=751065) | - | *Agaricomycetes* sp. | 257 | 2.00E-67 | 91.19 | Atheliaceae sp. |
| **OTU794** | 10.3864 | 0.0013 | 0.0403 | 1.0000 | 0.0 | 2.2 | NR:077067 | [SH1516571.08FU](https://unite.ut.ee/sh/SH1516571.08FU#fndtn-panel1) | *Debaryomyces prosopidis* | 357 | 2.00E-97 | 100 | *Debaryomyces* sp. |
| **OTU710** | 10.1501 | 0.0014 | 0.0443 | 1.0000 | 0.1 | 2.5 | [KJ416261](https://unite.ut.ee/bl_forw.php?id=489219) | [SH1512130.08FU](https://unite.ut.ee/sh/SH1512130.08FU#fndtn-panel1) | *Gymnopus ceraceicola* | 326 | 9.00E-88 | 86.8 | *Gymnopus* sp. |
